# Surface Anchoring and Active Sites of [Mo_3_S_13_]^2–^ Clusters as Co-Catalysts for Photocatalytic Hydrogen Evolution

**DOI:** 10.1021/acscatal.2c00972

**Published:** 2022-05-20

**Authors:** Samar Batool, Sreejith P. Nandan, Stephen Nagaraju Myakala, Ashwene Rajagopal, Jasmin S. Schubert, Pablo Ayala, Shaghayegh Naghdi, Hikaru Saito, Johannes Bernardi, Carsten Streb, Alexey Cherevan, Dominik Eder

**Affiliations:** † Institute of Materials Chemistry, TU Wien, Getreidemarkt 9/BC/02, 1060 Vienna, Austria; ‡ Institute of Inorganic Chemistry I, Ulm University, Albert-Einstein-Allee 11, 89081 Ulm, Germany; § Institute for Materials Chemistry and Engineering, Kyushu University, 6-1 Kasugakoen, Kasuga, Fukuoka 816-8580, Japan; ∥ University Service Centre for Transmission Electron Microscopy (USTEM), TU Wien, Wiedner Hauptstraße 8-10, 1040 Vienna, Austria; # Department of Chemistry, Johannes Gutenberg University Mainz, Duesbergweg 10-14, 55128 Mainz, Germany

**Keywords:** thiometalates, metal sulfides, heterogenization, water splitting, photocatalysis

## Abstract

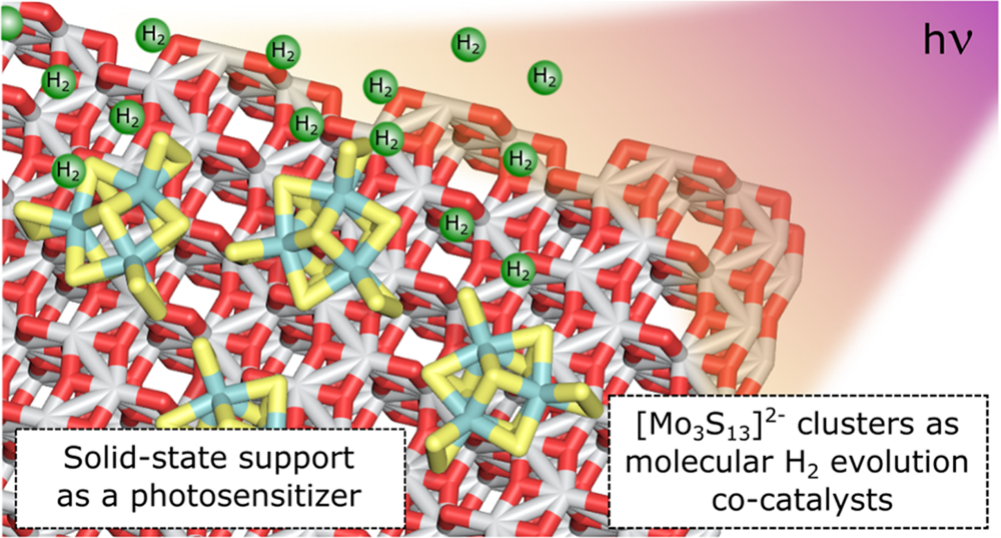

Achieving light-driven splitting of water with high efficiency remains a challenging task on the way to solar fuel exploration. In this work, to combine the advantages of heterogeneous and homogeneous photosystems, we covalently anchor noble-metal- and carbon-free thiomolybdate [Mo_3_S_13_]^2–^ clusters onto photoactive metal oxide supports to act as molecular co-catalysts for photocatalytic water splitting. We demonstrate that strong and surface-limited binding of the [Mo_3_S_13_]^2–^ to the oxide surfaces takes place. The attachment involves the loss of the majority of the terminal S_2_^2–^ groups, upon which Mo–O–Ti bonds with the hydroxylated TiO_2_ surface are established. The heterogenized [Mo_3_S_13_]^2–^ clusters are active and stable co-catalysts for the light-driven hydrogen evolution reaction (HER) with performance close to the level of the benchmark Pt. Optimal HER rates are achieved for 2 wt % cluster loadings, which we relate to the accessibility of the TiO_2_ surface required for efficient hole scavenging. We further elucidate the active HER sites by applying thermal post-treatments in air and N_2_. Our data demonstrate the importance of the trinuclear core of the [Mo_3_S_13_]^2–^ cluster and suggest bridging S_2_^2–^ and vacant coordination sites at the Mo centers as likely HER active sites. This work provides a prime example for the successful heterogenization of an inorganic molecular cluster as a co-catalyst for light-driven HER and gives the incentive to explore other thio(oxo)metalates.

## 1 Introduction

The ever-increasing energy consumption by our society leads to the unprecedented need for green and renewable fuels.^1^ With its high energy density, hydrogen can be seen as a suitable alternative to gasoline and natural gas;^2^ however, still today it is mostly generated from fossil fuels by steam reforming. Since the ratification of the Paris Agreement, alternative methods of hydrogen generation from water have attracted unprecedented attention including those through electrolysis using solar or nuclear power (*i.e.*, yellow and pink hydrogen). Among others, photocatalysis is seen as an ultimate sustainable solution that allows direct generation of hydrogen from renewable sources, such as water and sunlight. However, the efficiencies of contemporary heterogeneous photocatalytic systems are still far from the level to contribute substantially to the world energy demand.^3^ One important issue that requires urgent attention is the design of earth-abundant and high-performance co-catalyst able to facilitate the desired redox reaction at the photocatalyst surface.

Among various alternatives to noble-metal-based hydrogen evolution reaction (HER) catalytic systems,^4,5^ transition-metal chalcogenides—especially those from the MoS_2_ family—have shown excellent promise for electrochemical H_2_ production due to the presence of suitable adsorption and catalytic sites.^6^ After the realization that the basal planes of MoS_2_ are mostly inactive toward HER,^7−9^ a class of molecular thiomolybdate clusters^10,11^ that mimic the edge sites of MoS_2_ has gained interest for the applications in energy conversion. Over the past decade, [Mo_3_S_4_]^4+^,^12,13^ [Mo_3_S_13_]^2–^,^14−16^ [Mo_2_S_12_]^2–^,^17^ and their analogues^18−20^ have demonstrated exceptional electrochemical H_2_ generation (in terms of stability and low overpotentials) associated with the presence of abundant and accessible sulfur sites in their molecular structure. Compared to typical inorganic catalysts reported elsewhere,^21^ such clusters feature well-defined molecular structures, compositions, and geometries, which may further allow for in-depth studies and understanding of the active sites, reaction mechanisms, and dynamic nature of the (photo)catalytic processes.^22^ The implementation of these clusters in photocatalytic applications has so far been limited to several examples. On one hand, parallel to each other, two groups unraveled activity of [Mo_2_S_12_]^2–^ and [Mo_3_S_13_]^2–^ toward HER under strictly homogeneous conditions, when photosensitized by a molecular [Ru(bpy)_3_]^2+^ dye.^23,24^ On the other hand, two early studies have explored composites of [Mo_3_S_13_]^2–^ on Bi_2_WO_6_ and TiO_2_ for the application in light-driven degradation of methylene blue and acetone, respectively.^25,26^ Only a few studies so far have employed [Mo_3_S_13_]^2–^ as a dedicated HER co-catalyst in a combination with non-oxide supports,^27−29^ however, mainly relying on weak electrostatic interactions between the two components. To the best of our knowledge, none of these studies present detailed insights into the support/cluster attachment modes, structural modifications and, most importantly, the catalytic sites of these HER-relevant clusters after immobilization. Beside this, in light of the most recent record-breaking solar-to-hydrogen conversion efficiencies achieved on oxide-based semiconductors,^30−32^ exploration of the thiomolybdate–oxide interface and binding constitutes a highly relevant yet underexplored research subject.

Motivated by these factors, here we construct and investigate a set of promising earth-abundant photocatalysts comprising various photoactive oxide supports and [Mo_3_S_13_]^2–^ as a model HER co-catalyst. We show that the clusters undergo strong and irreversible covalent binding to the model TiO_2_ surface *via* Mo–O–Ti bond formation with the surface-hydroxyl groups, and that this surface anchoring is limited to monolayer formation and involves oligomerization of the cluster cores at high [Mo_3_S_13_]^2–^ surface density. We demonstrate a stable photocatalytic performance of the heterogenized [Mo_3_S_13_]^2–^ toward HER as a function of the loading with an optimal value of around 2 wt %, unravel factors limiting the performance at higher [Mo_3_S_13_]^2–^ coverage, and elaborate on the active state of the [Mo_3_S_13_]^2–^ under turnover conditions. Finally, we investigate the impact of the cluster structure and integrity on photocatalytic activity by subjecting it to dedicated heat treatments. Our results show that both the molecular structure of the Mo_3_ core and the presence of the bridging S_2_^2–^ ligands are key factors, enabling these clusters to act as efficient HER co-catalysts.

## 2 Results and Discussion

### 2.1 Cluster Preparation

**Figure 1:**
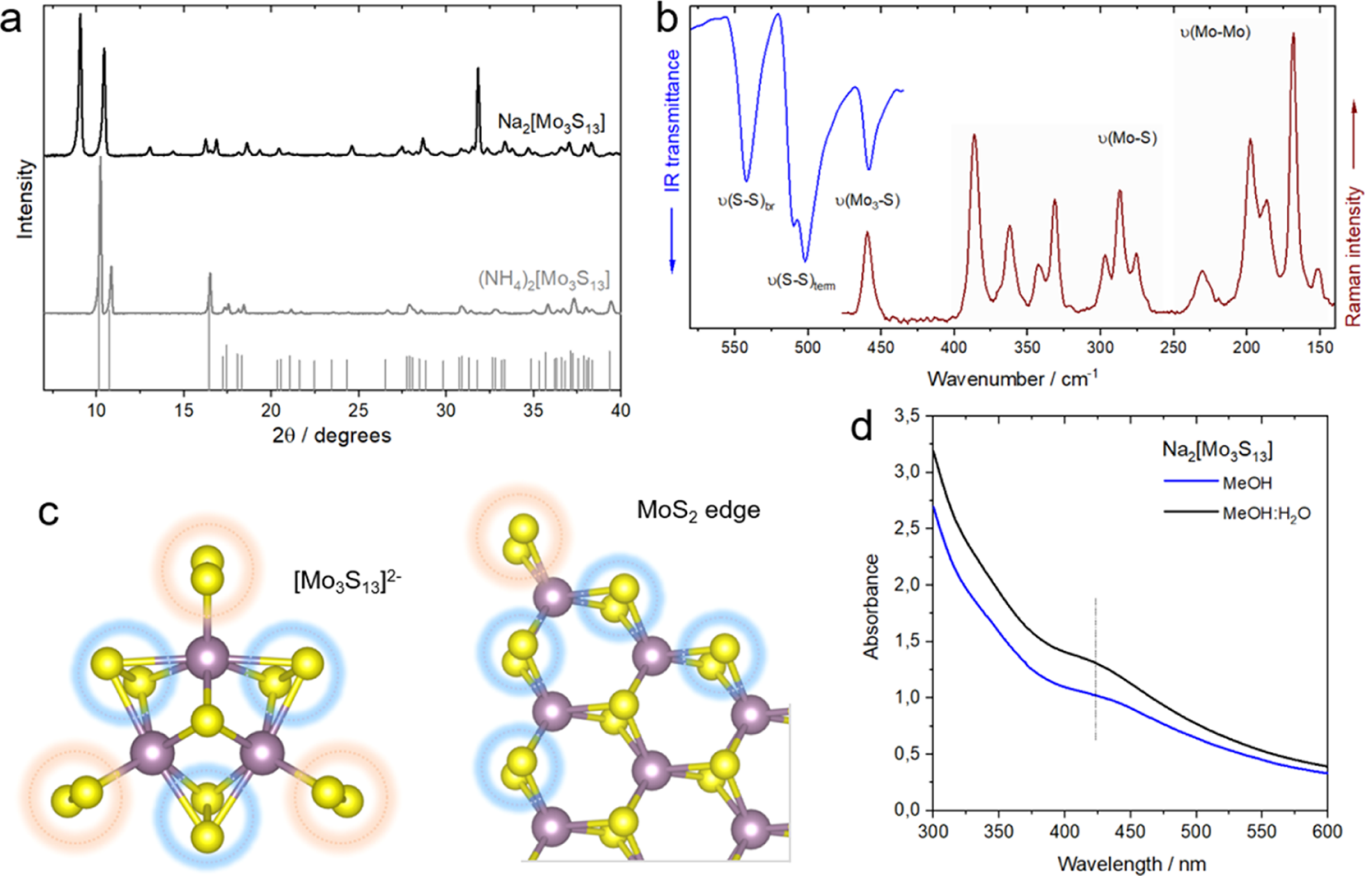
Cluster structure. (a) Powder XRD pattern of the Na_2_[Mo_3_S_13_] and (NH_4_)_2_[Mo_3_S_13_] along with the ICDD 04-021-7028 reference pattern of the ammonium salt, (b) overlayed ATR-FTIR and Raman spectra of the Na_2_[Mo_3_S_13_] powder featuring characteristic molecular vibrations and the corresponding ranges, (c) molecular model of the [Mo_3_S_13_]^2–^ compared to the edge structure of the MoS_2_ sheet, similar bonding motifs are highlighted; and (d) UV–Vis absorption spectrum of the 0.025 mM Na_2_[Mo_3_S_13_] solution in water and water/methanol featuring a characteristic absorption band.

(NH_4_)_2_[Mo_3_S_13_] and Na_2_[Mo_3_S_13_] were synthesized following reported procedures with minor modifications (see details in the experimental section). Their molecular and crystal structures were verified by a combination of spectroscopic, elemental, and morphological analyses (see details in Supporting Information, Section 1). X-ray diffraction (XRD) patterns of the as-prepared thiomolybdate salts indicate high crystallinity compounds (Figure 1a) and match well with the database and the data reported previously.^29^ SEM of the Na_2_[Mo_3_S_13_] shows rodlike crystals, typical of the sodium salt (Figure S1). ATR-FTIR spectroscopy reveals signature peaks centered at 542 cm^–1^, 505 (510/501 doublet) cm^–1^, and 458 cm^–1^, corresponding to bridging, terminal, and apical sulfur ligands, respectively (Figures 1b and S2).^29^ Complementary Raman spectra (Figure 1b) indicate additional peaks in 400–250 and 210–150 cm^–1^ ranges, which are characteristic of Mo–S and Mo–Mo vibrations of the [Mo_3_S_13_]^2–^ anion.^33,34^ The presence of Mo–Mo bonding is in line with the relatively short intermetallic distances in the cluster.^10^ Overall, the data confirm the trinuclear nature of the [Mo_3_S_13_]^2–^ and the presence of S^2–^/S_2_^2–^ ligands that make it structurally reminiscent of the MoS_2_-bonding motif (Figure 1c).

Ion exchange from NH_4_^+^ to Na^+^ (see the experimental section) renders the compound water and alcohol soluble (see details in Supporting Information, methods), which allows circumventing the high-boiling point dimethylformamide for further deposition and application in water splitting reactions. UV–vis spectra of Na_2_[Mo_3_S_13_] aqueous solution (0.025 mM) reveal an absorption centered at 417 nm (Figure 1d) corresponding to that of the powdered sample evaluated by diffuse-reflectance spectroscopy (DRS) in the solid state (Figure S3). Based on the electronic structure of the ammonium salt^35^ and the disappearance of this absorption band after oxidation (Figure S3), this characteristic absorption can be assigned to the (S_2_^2–^)_term_ → d (Mo) ligand-to-metal charge transfer (LMCT) transitions within the Mo-(S_2_) moiety.

### 2.2 Cluster Anchoring

After confirming the structure of the targeted Na_2_[Mo_3_S_13_] compound, we proceeded with cluster immobilization onto the model photocatalytic TiO_2_ surface following a wet-impregnation route (details in Experimental Section). A set of [Mo_3_S_13_]/TiO_2_ composites further denoted as xMo_3_/TiO_2_ was prepared with *x*—nominal cluster loading—ranging from 0.1 to 20 wt %.

#### 2.2.1 Loading and Dispersion

**Figure 2:**
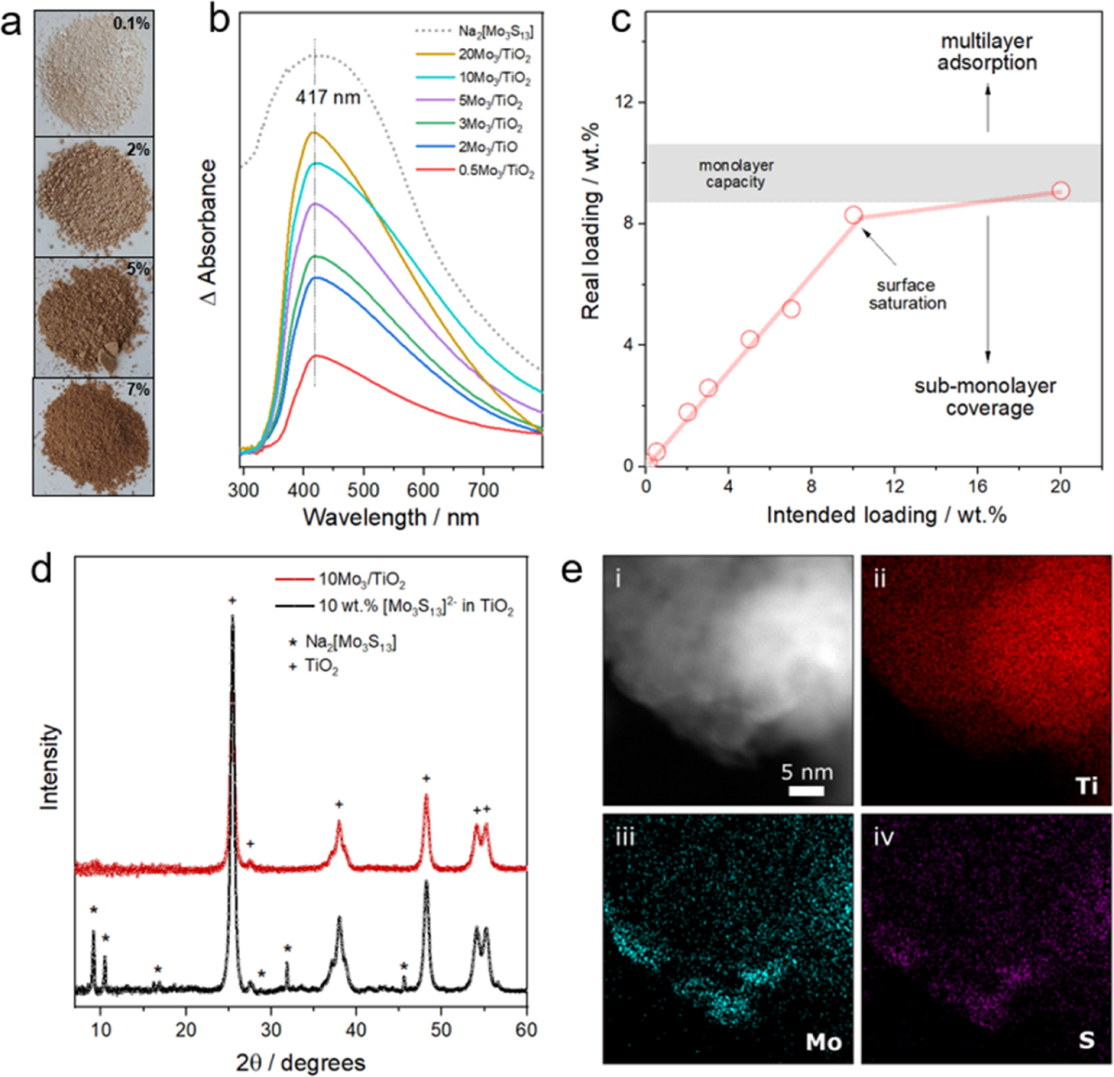
Cluster immobilization. (a) Digital photographs of the *x*Mo_3_/TiO_2_ samples with 0.1, 2, 5, and 7 wt % cluster loadings, (b) difference DRS spectra of the Mo_3_/TiO_2_ composites with different cluster loadings (0.1–20 wt %) subtracted from TiO_2_ absorption (see details in Supporting Information, methods) along with the DRS spectrum of Na_2_[Mo_3_S_13_] (dashed line), (c) real *vs* intended loading plot depicting the range of theoretical monolayer capacity (see details in Supporting Information, Section 6), a linear increase between the real and intended loadings for loadings <9 wt % and its saturation at higher loadings. (d) XRD pattern of Mo_3_/TiO_2_ composites (10 wt % loading) and of a physical mixture of Na_2_[Mo_3_S_13_] and TiO_2_ (1:9 wt %), (e) EDS-derived elemental mappings of Ti (ii), Mo (iii), and S (iv) in an exemplary 3Mo_3_/TiO_2_ composite.

The color of the Mo_3_/TiO_2_ composite series corresponds well to the increasing mass fraction of the [Mo_3_S_13_]^2–^ used for the synthesis (Figure 2a). DRS of the samples allows for a quantitative assessment and—after subtracting TiO_2_ spectra—shows a gradual increase of the characteristic LMCT band for higher cluster loadings (Figure 2b). However, a certain non-linearity of the trend can be seen at higher nominal loading values (Figure S4), which suggests an adsorption-limited process. Since our synthetic protocol involves extensive washing steps to remove loosely attached clusters, total reflection X-ray fluorescence (TXRF) spectroscopy was used to unravel the real [Mo_3_S_13_]^2–^ loadings in the composites.

The data (Table 1) reveal a close-to-linear increase in the cluster content (*i.e.*, real loadings correspond well to the nominal loadings) up to around 9 wt % at which saturation is achieved (Figure 2c). Based on the cluster footprint and surface area of the used TiO_2_ (116 m^2^/g, Figure S5), we estimate the theoretical adsorption capacity of our support (corresponding to a dense monolayer) to be around 9.6 wt % (see details in Supporting Information, Section 6). This value is close to that obtained experimentally, which strongly suggests that [Mo_3_S_13_]^2–^ cluster adsorption follows a monolayer formation and is thus limited by the surface area of the support. The complementary XRD pattern of the 10Mo_3_/TiO_2_ powder shows no sign of [Mo_3_S_13_]^2–^ compound (Figure 2d), which corroborates the molecular dispersion of the clusters on the support surface and excludes strong cluster aggregation (Figure S6 and Supporting Information, Section 7).

**Table 1:** Comparison between Intended (Nominal) [Mo_3_S_13_]^2–^ Loadings and Those Found in the As-Prepared [Mo_3_S_13_]/TiO_2_ Samples by Means of TXRF Quantification of Mo Content

nominal [Mo_3_S_13_]^2–^ loadings (wt %)	real [Mo_3_S_13_]^2–^ loadings (wt %)
0.1	0.14 ± 0.01
0.5	0.50 ± 0.05
2.0	1.78 ± 0.18
3.0	2.63 ± 0.26
5.0	4.22 ± 0.42
7.0	5.22 ± 0.52
10.0	8.30 ± 0.83
20.0	9.10 ± 0.91

Elemental maps acquired on the nanoscale through energy-dispersive X-ray spectroscopy (EDS) further confirm the homogeneous dispersion of Mo and S elements over the TiO_2_ surface (Figure 2e); however, some areas with locally higher Mo/S concentration can also be observed (Figure S7). Close examination of the Mo_3_/TiO_2_ composites with high-resolution transmission electron microscopy (HRTEM, Figure S8) reveals that while the majority of TiO_2_ surfaces seem to be smooth and intact, the areas of higher Mo and S content display structural ordering (up to a few layers), which may correspond to a certain degree of stacking or polymerization of the [Mo_3_S_13_]^2–^ clusters due to their proximity and dense packing at higher loading values.

#### 2.2.2 Surface Structuring

**Figure 3:**
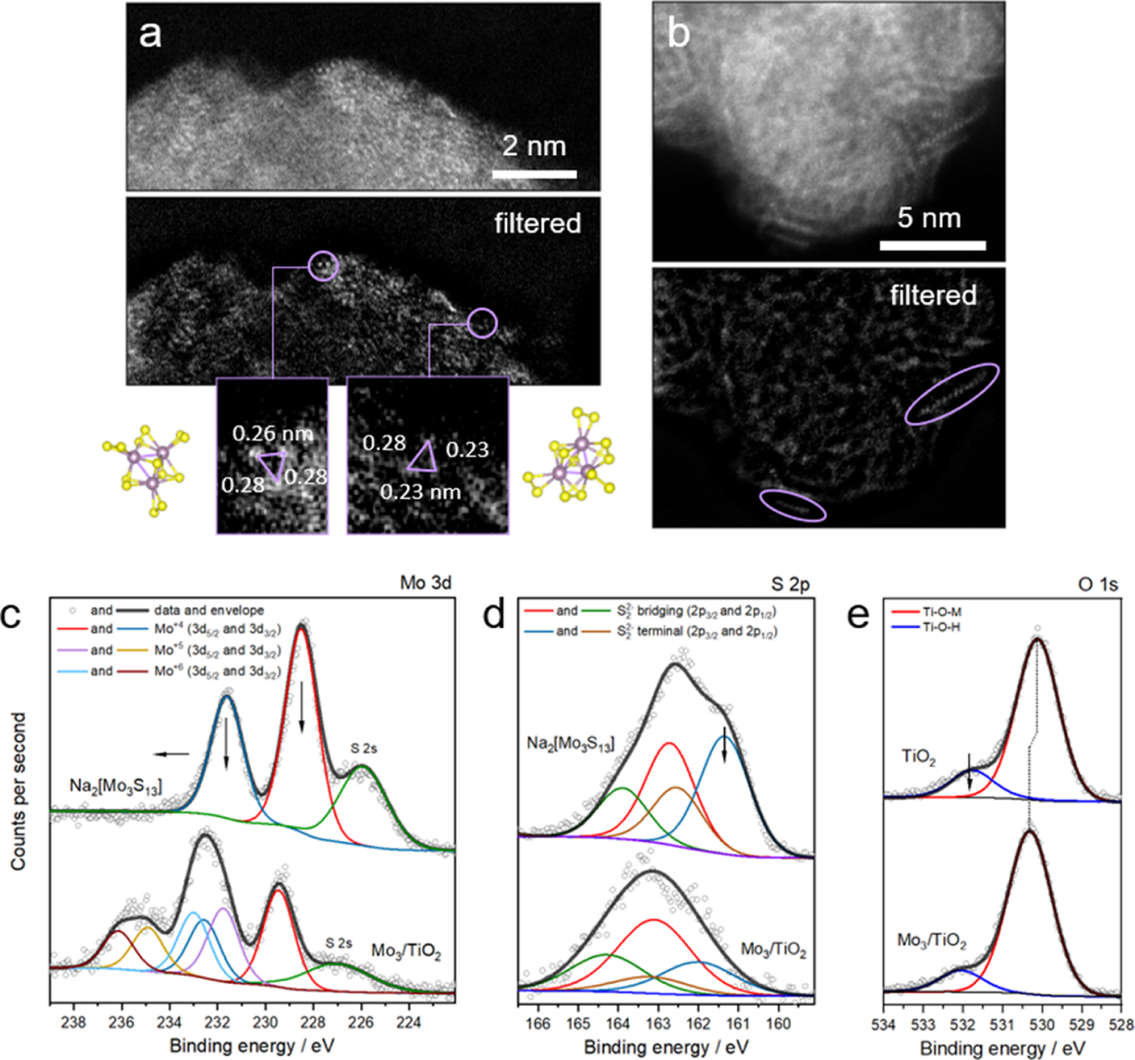
Cluster attachment. (a,b) HAADF-STEM images of 3Mo_3_/TiO_2_ composites; Fourier filtered images are shown in the bottom panels; examples of detected {Mo_3_} cores are circled and magnified in the insets in (a), where they are compared with the model of tilted [Mo_3_S_13_]^2–^ cluster cores, (c–e) XPS spectra of the Na_2_[Mo_3_S_13_] and the clusters after attachment to the TiO_2_ surface (c) Mo 3d region, (d) S 2p, and (e) O 1s regions with corresponding fits; real [Mo_3_S_13_]^2–^ loading is derived *via* TXRF to be 2 wt %.

Aberration-corrected high-angle annular dark-field scanning transmission electron microscopy (HAADF-STEM) was employed to provide an atomistic view on the surface-anchored clusters. Figure 3a shows a high-resolution micrograph of the 3Mo_3_/TiO_2_ sample and reveals a collection of bright spots distributed over the edge of an individual support nanoparticle. Based on the Z-contrast difference between Ti and Mo, along with the observed spot size smaller than 1 Å, each spot likely corresponds to an individual Mo atom, wherein the Mo–Mo distance of 2.8 Å and less (due to the tilted position of the clusters on the surface) could be measured, in line with that within the [Mo_3_S_13_]^2–^ cluster core (2.72 Å). Depending on the orientation of the supported clusters (insets are shown in Figure 3a), we can identify a number of triangular formations, which suggests the intact structure of the {Mo_3_} cluster cores upon binding, in line with their high structural stability.^36^Figure 3b shows a high-resolution image of an area with relatively higher Mo/S concentrations according to EDS signal. It reveals an assembly of bright spots organized in chain-like meta-structures, as highlighted by circled regions in the filtered image. The estimated distance between the spots of 2.7 ± 0.4 Å strongly suggests that the chains correspond to oligomers made of Mo atoms originated from the {Mo_3_} cores. Similar to the formation of two-dimensional MoS_*x*_ nanostructures from [Mo_3_S_13_]^2–^ on graphite reported previously,^18^ our HAADF-STEM data thus suggest that cluster polymerization—likely *via* partial loss and sharing of terminal disulfide ligands—takes place also in the case of the oxide support, however only at high cluster densities. The restructuring to chain-like structures shows the tendency of the clusters to organize in a highly disordered MoS_2_-like motif similar to that described recently.^37^

#### 2.2.3 Adsorption Model

To elucidate the specificity and strength of the cluster anchoring, we set up a series of impregnation experiments using concentrated Na_2_[Mo_3_S_13_] solutions and a range of alternative substrates including anatase TiO_2_ with a significantly larger particle size and rutile TiO_2_ and BiVO_4_ powders (see details in Supporting Information, Section 10). Figure S9 plots real cluster loadings (quantified through TXRF) and surface areas of the supports used (Table S1). The trend reveals a strong dependency between the two parameters: while only 0.19 wt % of [Mo_3_S_13_]^2–^ anchored onto low-surface area BiVO_4_ (1.11 m^2^/g), 5.14 wt % of [Mo_3_S_13_]^2–^ could be accommodated by the surface of rutile nanopowder (27 m^2^/g). Based on these data, the surface-limited [Mo_3_S_13_]^2–^ deposition seems to be independent of the composition of the chosen oxides, which allows us to suggest that the formation of [Mo_3_S_13_]^2–^ monolayer involves irreversible chemical bonding (chemisorption) with the support surface. The high strength and durability of the [Mo_3_S_13_]^2–^ anchoring were further corroborated in a set of leaching experiments (see details in Supporting Information, Section 11), which overall strongly suggests that other semiconducting oxide-based materials will also act as suitable supports for the cluster deposition.

#### 2.2.4 Binding Modes

The data conclusively show that the attachment of [Mo_3_S_13_]^2–^ onto oxide surfaces is irreversible and surface-limited. For the series of Mo_3_/TiO_2_ composites based on anatase nanoparticles (116 m^2^/g), the maximum achieved [Mo_3_S_13_]^2–^ loadings of 9.1 wt % thus correspond to a dense monolayer coverage. Confirmation of the cluster integrity after immobilization *via* Raman/ATR-FTIR, however, renders challenging as only a weak set of characteristic Mo–S, S–S, and Mo–Mo vibrations could be seen in the 20Mo_3_/TiO_2_ sample, that is, with the highest cluster loading (Figure S10).

Therefore, we employed surface-sensitive X-ray photoelectron spectroscopy (XPS) to verify the cluster structure and elucidate their binding modes to the oxide support (see details in Supporting Information, methods). Quantification of the relative Mo-to-S ratios from survey spectra (Figure S11) reveals a strong relative loss of S (around 50%) upon anchoring (Tables S2 and S3), which provides a first hint for the binding scenario. Detailed analyses of the Mo 3d edge (Figure 3c) show that partial oxidation of Mo (original oxidation state +4) to a mixture of +5/+6 takes place, while the change in the S 2p edge profile (Figure 3d) implies that terminal disulfide ligands are mostly affected by the anchoring process. Considering the additional shift of Mo signals to higher binding energy, the overall data suggest that [Mo_3_S_13_]^2–^ loses more labile terminal ligands and establishes a covalent binding with the strong electron-withdrawing hydroxyl groups of the oxide surface, likely forming Mo–O–Ti bonds. In line with this assumption, XPS data of the O 1s edge (Figure 3e) indicate a noticeable shift of the prime O signal (Ti–O–Ti) to higher binding energies (530.1 eV for TiO_2_ to 530.3 eV for Mo_3_/TiO_2_) accompanied by a decrease in surface-hydroxyl groups by 5% (see details in Supporting Information, Section 13.2). Both observations corroborate the transformation of Ti–OH groups into Ti–O–Mo. Observed shifts in binding energy values further indicate that a considerable degree of electron density flows from the [Mo_3_S_13_]^2–^ to the titania support, as expected from the anionic charge of the clusters (see details in Supporting Information, Section 13.2). Overall, XPS data show that terminal S_2_^2–^ groups get replaced upon anchoring to allow for covalent binding with TiO_2_. Although most of the clusters lose their original [Mo_3_S(S_2_)_3_^term^(S_2_)_3_^bridg^]^2–^ composition, the integrity of their tri-nuclear {Mo_3_(μ-S_2_)_3_} cores can be confirmed.^38^ A certain degree of oligomerization of the {Mo_3_} cores *via* remaining terminal disulfides, however, cannot be excluded based on our STEM data.

### 2.3 Photocatalytic Performance

The set of the prepared *x*Mo_3_/TiO_2_ composites allows us to evaluate the prospected co-catalytic function of the [Mo_3_S_13_]^2–^ clusters toward HER and—considering the structural changes upon binding—can further provide relevant information regarding their active sites. Several reports have attempted to identify catalytic HER sites of thiometalate clusters by examining their electrocatalytic HER performance. Based on DFT calculations and experimental evidence, the groups of Joh,^16^ Miras,^19^ and Beyer^39,40^ agree that terminal sulfides are the preferred sites for hydrogen adsorption and the most favorable catalytic sites for electrochemical HER. In contrast to this, the group of Artero observed a loss of terminal disulfides under the turnover conditions and thus suggested the unsaturated Mo centers to act as catalytic sites.^18^ Joo and colleagues corroborated this idea and identified Mo-oxo species to play a key role in generating effective hydrogen adsorption sites.^20^ Complementary to these, the group of Streb recently examined the photocatalytic performance of the [Mo_3_S_13_]^2–^ under homogeneous conditions.^22^ They revealed a dynamic structure of the cluster that involves the partial exchange of terminal disulfides with aqua ligands under turnover conditions. All three groups, however, agree that the formation Mo–H or Mo=O intermediate could be possible when Mo centers with undercoordinated sites are present in the system, which is the case for our attachment model.

#### 2.3.1 Clusters as HER Co-Catalysts

**Figure 4:**
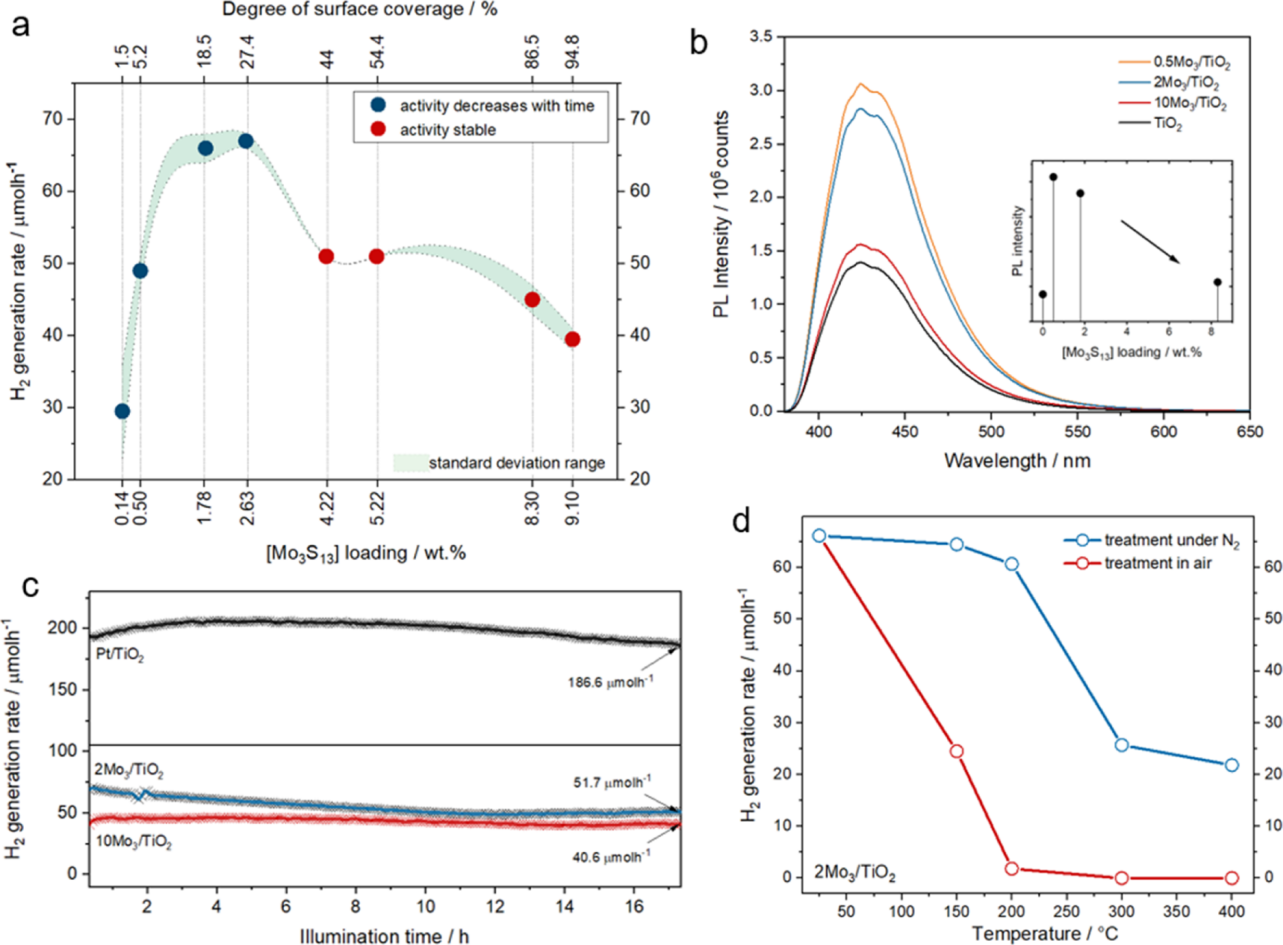
Photocatalytic performance and active sites. (a) Hydrogen evolution rates plotted against the [Mo_3_S_13_]^2–^ cluster content and the degree of surface coverage in %; green area shows the standard deviation of hydrogen evolution rate values obtained from multiple measurements for each loading value, (b) PL spectra obtained from the catalyst solutions containing TA as OH trap after UV pre-illumination; inset shows intensities of the peak maximum (*ca.* 425 nm corresponding to hydroxyterephthalic acid emission) plotted against the real [Mo_3_S_13_]^2–^ loadings, (c) long-term photocatalytic hydrogen evolution experiments of Mo_3_/TiO_2_ composites with 2 and 10 wt % loadings and their comparison with Pt/TiO_2_ in terms of HER stability, (d) comparison of the hydrogen generation rate of Mo_3_/TiO_2_ composites heat-treated (25–400 °C) under air and the N_2_ atmosphere; full HER profiles are shown in Figures S12 and S19.

The as-obtained short-term H_2_ evolution profiles of the Mo_3_/TiO_2_ composites are shown in Figure S12a (experimental section for setup and reactor description and Figure S12b for reproducibility). Figure 4a plots the extracted HER rates against cluster loading (real values from Table 1 are considered) and surface area coverage (theoretical values assuming monolayer adsorption are considered). Interestingly, from the point of view of the proposed monolayer adsorption model, the HER performance of the Mo_3_/TiO_2_ photocatalysts follows a volcano trend: the rate of H_2_ evolution peaks at around 2 wt % value and drops gradually for higher cluster loadings. We can exclude a significant contribution of the [Mo_3_S_13_]^2–^ parasitic absorption^29^ to the HER decline (Figure 2b) based on the sufficiently high light flux used in our experiments (Figure S13). However, this HER trend can be explained by considering the overall redox cycle: while higher [Mo_3_S_13_]^2–^ loadings correspond to more active HER sites, they may diminish the extent of the available TiO_2_/solution interface necessary for an efficient hole scavenging.^41^ A set of radical trapping photoluminescence (PL) experiments using terephthalic acid (TA) as a probe molecule were performed to elaborate on this point (see details in the experimental section). As depicted in Figure 4b, compared to neat TiO_2_, we observe a significant increase in OH radical generation on Mo_3_/TiO_2_ samples, especially at lower cluster contents. This result corresponds to the enhanced separation of photogenerated charge carriers and manifests the ability of the thiomolybdate clusters to extract the electrons from the support, in line with their role as HER co-catalysts. The inset in Figure 4b, however, illustrates that higher cluster loadings (*i.e.*, 10Mo_3_/TiO_2_) restrict the ability of Mo_3_/TiO_2_ to form OH radicals. As a consequence, the inefficient utilization of photogenerated holes leads to the acceleration of the recombination rates. These data validate our assumption and confirm that the extent of the available TiO_2_/solution interface becomes a factor that limits the overall HER performance at higher cluster loading values.

In addition to the overall HER trend, Figure 4a reveals that the composites with the cluster content below 3 wt % show mild deactivation (*i.e.*, decrease in the H_2_ evolution rate with time, Figure S12a) over the first 60 min of illumination, while those with the [Mo_3_S_13_]^2–^ content above 3 wt % exhibit stable HER performance. This result hints toward a coverage-dependent stabilization of {Mo_3_S_6_} cores and their active sites, which may be related to the oligomerization of the closely packed clusters under turnover conditions.^18^ Lastly, both types of composites feature robust H_2_ evolution over a long-term reaction (Figure 4c). The performance of 10Mo_3_/TiO_2_ can even be compared to the benchmark HER photocatalyst couple of Pt on TiO_2_ tested under identical conditions (see details in Supporting Information, methods), which manifests these thiomolybdate clusters as efficient and stable noble-metal-free HER co-catalysts. The catalytic nature of H_2_ evolution can be further confirmed by comparing the number of H_2_ molecules generated (0.88 mmol) with the number of Mo atoms present in the exemplary 2Mo_3_/TiO_2_ photosystem (0.75 μmol).^42^

Post-catalytic characterization of the Mo_3_/TiO_2_ composites uncovers several key points. As revealed by EDS mapping (Figure S14a) and TXRF (Figure S14b) of the catalyst recovered after HER, we observe neither change in Mo distribution nor leaching of the Mo content from the TiO_2_ surface. This is in strong agreement with the stable HER rates discussed before. Detailed XPS spectra, however, show that a mild transformation of the clusters upon turnover conditions takes place: following the oxidation of Mo centers and the loss of terminal S_2_^2–^ upon cluster anchoring (see previous discussions), even more of the Mo^4+^ turns into Mo^5+/6+^ (Figure S15a), while a part of bridging S_2_^2–^ disappears from the structure (Figure S15b). Both changes can be indicative of the active HER state of the clusters and are in line with the dynamic exchange of the disulfide ligands with aqua/hydroxo ligands under catalytic conditions.^22^

#### 2.3.2 Active Sites

In order to verify the impact of the molecular composition and structure of [Mo_3_S_13_]^2–^ on its HER activity, we subjected the as-prepared Mo_3_/TiO_2_ photocatalysts to a series of heat treatments. Thermogravimetric analysis (TGA, Figure S16) performed in air and N_2_ reveal that the clusters undergo structural changes in the temperature range of 250–450 °C. Earlier reports suggested that apical S^2–^ is most thermolabile,^36^ while terminal and bridging disulfides require higher temperature for decomposition/restructuring.^16^ Moreover, according to *in situ* XRD, heating of [Mo_3_S_13_]^2–^ in N_2_ yields 2H–MoS_2_ (Figure S17),^43^ while calcination under ambient air ultimately results in the formation of MoO_3_ (Figure S18). Importantly, for the following discussion, heat treatments in air tend to degrade the clusters more rapidly (*i.e.*, at lower temperatures) due to facilitated ligand oxidation.

HER tests were performed on 2Mo_3_/TiO_2_ after respective heat treatments in air and N_2_ up to 400 °C (full HER profiles in Figure S19). Figure 4d shows that the H_2_ evolution rates start to decline in both cases after a certain temperature is reached. Importantly, for the treatments in air, the activity drops sharply—close to zero—already at 200 °C, which is likely related to the oxidation (ligand cleavage) of the {Mo_3_(μ-S_2_)_3_} cores facilitated by the O-rich support surface *via* the Mars-van Krevelen mechanism.^44,45^ In the case of N_2_-treated samples, the activity is unaffected at 200 °C but drops strongly—by 60%—when 300 °C treatment is applied. Both trends conclusively show that the molecular composition of the cluster and the presence of sulfur ligands in its original structure are crucial structural factors that allow for high HER performance.

In conjunction with our observation that the majority of terminal S_2_^2–^ ligands tend to be displaced upon [Mo_3_S_13_]^2–^ attachment, these activity trends correlate well with previous reports,^18,20,22^ suggesting that both accessible Mo centers (either undercoordinated or oxo species) and bridging S_2_^2–^ ligands constitute essential structural motifs, rendering these clusters as high-performance and stable HER co-catalysts.^46^ The contribution of the remaining terminal S_2_^2–^ as proton adsorption or catalytic HER sites can, however, not be entirely excluded considering their persistent presence in the Mo_3_/TiO_2_ catalyst before and after catalysis. Finally, in light of our post-catalytic studies, the active HER state of the [Mo_3_S_13_]^2–^ formed under turnover conditions seem to involve a more complex and dynamic interplay between available Mo sites, partially replaced ligands, and oligomerized {Mo_3_} cores; dedicated operando studies will be required to unravel individual contributions of these factors.

## 3 Conclusions

In this contribution, we demonstrate the immobilization of an all-inorganic thiomolybdate [Mo_3_S_13_]^2–^ cluster on various metal oxide surfaces and investigate its function as a co-catalyst for photocatalytic HER. The results indicate that the attachment of the [Mo_3_S_13_]^2–^ on TiO_2_ is strong and irreversible and that it follows monolayer adsorption, whereas the surface coverage is directly proportional to the cluster loadings. Elemental mappings confirm that the majority of the [Mo_3_S_13_]^2–^ species distribute homogeneously over the support surface. STEM-HAADF images allow us to resolve individual {Mo_3_} cores attached to the surface and also indicate the formation of chain-like structures presumably made of oligomerized cluster cores. Detailed XPS analyses show that the attachment involves partial oxidation of Mo centers and partial loss of the terminal S_2_^2–^ ligands, which we assign to the formation of covalent Ti–O–Mo bonds at the support–cluster interface. We further demonstrate stable, loading-depended HER performance of the prepared Mo_3_/TiO_2_ photocatalysis, which reaches an optimum at around 2–3 wt % cluster loading—value limited by the hole utilization efficiency. Post-catalytic studies confirm no leaching of the Mo species (in line with strong bonding) but strongly indicate a further milder transformation of the clusters upon turnover conditions involving ligand exchange. Finally, by subjecting our composites to stepwise thermal decomposition, we demonstrate that both the molecular structure of the Mo_3_ cores and the presence of the bridging S_2_^2–^ ligands in the parent structure are responsible for the excellent HER performance. This work serves as an important example of the implementation of [Mo_3_S_13_]^2–^ clusters as co-catalysts for photocatalytic applications and provides insights into their active states and structures, which will be of interest to other molecular systems and applications. Exploration of visible-light active supports and other all-inorganic clusters such as polyoxometalates^47^ is envisioned to develop tunable photosystems for efficient sunlight-driven generation of H_2_ and other solar fuels.

## 4 Experimental Section

A detailed overview of the used chemicals, analytical instrumentation, characterization methods, supplementary figures, and discussions are provided in the Supporting Information. Synthetic protocols and photocatalytic activity measurements are detailed below.

### 4.1 Synthesis of (NH_4_)_2_[Mo_3_(μ_3_-S^2–^)(μ,η^2^-S_2_^2–^)_3_(η^2^-S_2_^2–^)_3_]·H_2_O

The precursor for the synthesis of Na_2_[Mo_3_S_13_]·H_2_O was prepared using a modified procedure reported by Müller *et al.*(48) A solution of (NH_4_)_2_[Mo_7_O_24_]·H_2_O (3.2 mmol) was prepared in water (20 mL) in a round-bottom flask followed by the addition of (NH_4_)_2_S_*x*_ (25 wt %, 120 mL). The resulting red-colored solution was heated at 96 °C for 5 days under continuous stirring. The dark red colored product was obtained by filtration and thoroughly washed with water, ethanol, carbon disulfide, and diethyl ether. The product was dried at 60 °C in the air (yield: 90%).

### 4.2 Synthesis of Na_2_[Mo_3_(μ_3_-S^2–^)(μ,η^2^-S_2_^2–^)_3_(η^2^-S_2_^2–^)_3_]·H_2_O

The sodium salt was synthesized following a reported method by Weber *et al.*(33) Briefly, 250 mg of (NH_4_)_2_[Mo_3_S_13_]·H_2_O was added to a 1% NaOH solution (40 mL) followed by stirring under vacuum for 2 h. The mixture was then filtered in 10% NaCl solution and kept for 12 h in order to precipitate out the desired Na_2_[Mo_3_S_13_]·H_2_O (yield: 70%).

### 4.3 Synthesis of Na_2_[Mo_3_(μ_3_-S^2–^)(μ,η^2^-S_2_^2–^)_3_(η^2^-S_2_^2–^)_3_]·H_2_O/TiO_2_ Composites

The Na_2_[Mo_3_S_13_]·H_2_O/TiO_2_ composites having different weight contents (0.1, 0.5, 2, 3, 5, 7, 10, and 20%) of thiomolybdate clusters were synthesized. TiO_2_ powder was dispersed in MeOH (100 mg in 28 mL) by ultrasonication for 10 min. The clusters (amount corresponding to the nominal loading) were dissolved in methanol, added to the TiO_2_ suspension, and again sonicated for 15 min. The mixture was then kept on stirring for 24 h to allow for adsorption, followed by filtration and repeated washing with methanol to remove unattached clusters and those attached loosely (*e.g.*, in a layer-by-layer fashion). Filtration and washing of the powders with lower intended cluster loadings (*e.g.*, below 10 wt % for TiO_2_) resulted in colorless filtrates. Filtration of the powders with higher intended loadings (*e.g.*, above 10 wt % for TiO_2_) gave colored filtrates, while washing was repeated until the filtrates turned colorless to ensure the removal of excess clusters. The final powders were dried at 60 °C and are denoted as *x*Mo_3_/TiO_2_ throughout the manuscript, where *x* stands for the nominal (intended) mass content of the clusters.

### 4.4 Photocatalytic Experiments

The hydrogen evolution experiments were carried out using a top-down irradiation gas-flow slurry type custom-built reactor (total volume of 100 mL) equipped with a monochromatic UV LED light source with an incident light intensity of 0.49 W centered at 365 ± 6 nm (196 mW/cm^2^, Thorlabs SOLIS). In the reaction setup, 10 mg of the powdered photocatalyst was introduced into the reactor containing 40 mL of 1:1 vol % MeOH/H_2_O mixture (activity *vs* catalyst mass curves are presented in Figure S13). The reaction mixture was dispersed evenly by ultrasonication for 10 s. During the experiment, the reactor was continuously purged with argon carrier gas at a flow rate of 30 mL min^–1^, which is controlled by a mass flow controller (MCC-instruments); the reaction solution was stirred at 500 rpm. The gaseous H_2_ was detected directly in the stream by an online gas analyzer (X-stream, Emerson Process Management) equipped with a thermal conductivity detector. H_2_ concentrations were deduced based on a multilevel calibration. The temperature of the reactor was maintained at 15 °C using a water-cooling system (Lauda). In a single experiment, the reaction mixture was stirred for 20 min before starting the illumination to attain a stable signal baseline, followed by a 60 min light-on cycle and a 40 min resting in the dark. A typical H_2_ evolution profile (*e.g.*, as shown in Figure S12) obtained with our flow reactor includes an “induction” period (increasing H_2_ evolution rate during the first 5–10 min) that is due to the fact the H_2_ gas first needs to fill the dead volume (*e.g.*, reactor volume and tubing volume) to reach the detector. After this “induction,” H_2_ evolution reaches a stable rate, which speaks for stable HER performance. In contrast, when the rate changes over time, (de)activation of the photocatalytic system can be deduced.^49,50^ When the illumination is stopped, the signal returns to its baseline.

### 4.5 PL Measurements

The photocatalytic mechanism was investigated using radical-trapping PL emission spectroscopy employing TA as an OH radical scavenger following earlier reports.^51^ In a single experiment, 1 mg/mL aqueous suspension of the catalyst (TiO_2_ and Mo_3_/TiO_2_ composites) was prepared and diluted with 3 × 10^–3^ M TA solution in 0.01 M NaOH. The suspension was illuminated for 40 min with UV light (for conditions, see above), followed by centrifugation at 5600 rpm for 30 min to separate the catalyst from the solution. PL emission of this solution was probed with an excitation wavelength of 315 nm (see details in Supporting Information, methods). According to the method, photoexcited holes generated during the illumination of the photocatalyst suspensions form OH radicals at the catalyst/solution interface; the OH radicals in the solution can be next effectively scavenged by the TA molecules, resulting in the formation of 2-hydroxyterephthalic acid (TA-OH). As TA-OH is highly fluorescent, PL can be used to quantify the amount of so-generated OH radicals and thus can be used to assess the extent of electron–hole separation and the effectiveness of hole utilization.

## Supporting Information Available

Extended experimental section, SEM images of Na_2_[Mo_3_S_13_], additional ATR-FTIR and DRS spectra, physisorption data, estimation of the theoretical surface coverage, extended XRD patterns and additional EDX maps and (S)TEM images of the Mo_3_/TiO_2_ samples, discussion of the alternative supports, cluster leaching experiments, Raman spectra, XPS data (quantification) along with the discussion of the cluster binding and interfacial charge transfer, photocatalytic data (HER profiles, reproducibility, and optimization), post-catalytic characterization of the Mo_3_/TiO_2_ photocatalysts (TXRF, EDS, and XPS), and thermal analyses of the [Mo_3_S_13_]^2–^ salts (TGA, *in situ* XRD) (PDF)
