# Supplementary material for: Surface Anchoring and Active Sites of [Mo3S13]2– Clusters as Co-Catalysts for Photocatalytic Hydrogen Evolution
Source: ACS Catal. 2022 May 20;12(11):6641–50. doi: 10.1021/acscatal.2c00972 (PMC9171716; doi:10.1021/acscatal.2c00972)
Supplement: Supplementary file 1 — cs2c00972_si_001.pdf [file cs2c00972_si_001.pdf]

Supporting Information (SI)

Surface-Anchoring and Active Sites of $[Mo_3S_{13}]^{2-}$ Clusters as Co-Catalysts for Photocatalytic Hydrogen Evolution

Samar Batool[1]†, Sreejith P. Nandan[1]†, Stephen Nagaraju Myakala[1], Ashwene Rajagopal[2], Jasmin S. Schubert[1], Pablo Ayala[1], Shaghayegh Naghdi[1], Hikaru Saito[3], Johannes Bernardi[4], Carsten Streb[2,5], Alexey Cherevan[1,] and Dominik Eder[1]*

[1] *TU Wien, Institute of Materials Chemistry, Getreidemarkt 9/BC/02, 1040, Vienna, Austria*

[2] *Ulm University, Institute of Inorganic Chemistry I, Albert-Einstein-Allee 11, 89081 Ulm, Germany*

[3] *Institute for Materials Chemistry and Engineering, Kyushu University, 6-1 Kasugakoen, Kasuga, Fukuoka 816-8580, Japan*

[4] *TU Wien, University Service Centre for Transmission Electron Microscopy (USTEM), Wiedner Hauptstraße 8-10, 1040, Vienna, Austria*

[5] *Department of Chemistry, Johannes Gutenberg University Mainz, Duesbergweg 10-14, 55128 Mainz, Germany*

[†] *these authors contributed equally*

* *corresponding author*

correspondence to alexey.cherevan@tuwien.ac.at

Content

1. List of chemicals and methods 3
2. SEM images 6
3. Additional ATR-FTIR spectra 6
4. Additional DRS spectra and analysis 7
5. BET measurements 8
6. Estimation of the theoretical surface coverage 9
7. Extended XRD patterns 10
8. Additional EDS maps 11
9. TEM images 12
10. Deposition of $[Mo_3S_{13}]^{2-}$ on alternative supports 13
11. Leaching experiments 14
12. Additional Raman spectra 15
13. XPS discussion 16
14. Supplementary photocatalytic performance data 18
15. Post-catalytic characterization 19
16. Thermal treatments (TGA and *in situ* XRD) and HER performance 20
17. List of references 22

1. List of chemicals and methods

Chemicals

All the chemicals used for the synthesis were purchased from commercial suppliers and include ammonium molybdate tetrahydrate ($(NH_4)_6[Mo_7O_{24}]\cdot 4H_2O$, Sigma-Aldrich, 99.98% trace metals basis), ammonium sulfide solution $(NH_4)_2S_x$, Sigma-Aldrich, 20 wt.% in H_2O), carbon disulfide (CS_2, Sigma-Aldrich, 99.9%), sodium chloride (NaCl, Carl Roth), sodium hydroxide (NaOH, Carl Roth), ammonium metavanadate (NH_4VO_3, Carl Roth, 99.8%), bismuth (III) nitrate pentahydrate ($Bi(NO_3)_3\cdot 5H_2O$, Riedel de Haen, 98%), titanium (IV) oxide anatase nanopowder (TiO_2, Sigma-Aldrich, 99.7%, <25 nm particle size) and titanium (IV) oxide rutile nanopowder (TiO_2, Sigma-Aldrich, 99.7%, <100 nm particle size). The solvents used for the synthesis were deionized water, ethanol (EtOH, from Chem-Lab NV), HPLC-gradient grade methanol (MeOH, from VWR), diethyl ether (from Sigma-Aldrich), N,N-dimethylformamide (DMF, from Acros Organics, 99.8%, extra dry over the molecular sieve, acroseal).

Characterization techniques

UV-Vis spectroscopy was performed on a Jasco V670 UV-Vis spectrometer. The samples were prepared in methanol and aqueous methanol (1:1 vol.) solution with a concentration of 0.025 mM; UV-Vis spectra were recorded in absorbance mode. Absorption spectra of powdered samples were measured in solid-state via diffuse-reflectance spectroscopy (DRS) using $MgSO_4$ as a reference. The Mo_3/TiO_2 composite samples were measured using TiO_2 as a background.

ATR-FTIR spectra of the samples were recorded via PerkinElmer FTIR Spectral UATR-TWO with a spectrum two Universal ATR (Single Reflection Diamond) instrument. Powdered samples were directly loaded onto the sample holder and the spectra were recorded in the region of 4000-400 wavenumbers (cm^{-1}). Raman measurements were performed with a WITec alpha 300 RSA+ Raman microscope equipped with a 632 nm excitation laser maintaining the laser intensity at 1 mW.

The quantitative elemental analysis of the samples was performed with X-ray photoelectron spectroscopy (XPS) using a custom-built SPECS XPS-spectrometer equipped with a monochromatized Al-K_α X-ray source (µ 350) and a hemispherical WAL-150 analyzer (acceptance angle: 60°). To improve the sensitivity of the measurements, Mo_3/TiO_2 samples were prepared and investigated in the form of thin-films (see Additional Methods in SI). This was followed by wet impregnation of the $Na_2[Mo_3S_{13}]$ clusters from methanolic solutions. For a single XPS measurement, a slide containing the sample film was placed on the sample holder using a conductive clip. Pass energies of 100 eV and 30 eV and energy resolutions of 1 eV and 100 meV were used for survey and detail spectra, respectively (excitation energy: 1486.6 eV, beam energy and spot size: 70 W onto 400 µm, angle: 51° to sample surface normal, base pressure: 5×10^{-10} mbar, pressure during measurements: 2×10^{-9} mbar). Data analysis was performed using CASA XPS software, employing transmission corrections (as per the instrument vendor's specifications), Shirley and Sh Tougaard backgrounds and Scofield sensitivity factors. Charge correction was applied so the adventitious carbon peak (C–C peak) was shifted to 284.8 eV binding energy (BE). All content values shown are in units of relative atomic percent (at.%), where the detection limit in survey measurements usually lies around 0.1–1 at.%, depending on the element.

Quantitative determination of the $[Mo_3S_{13}]^{2-}$ cluster loadings was performed by X-ray fluorescence spectroscopy in total reflection geometry (TXRF) using an ATOMIKA 8030C X-ray fluorescence analyzer (Atomika Instruments GmbH, Oberschleissheim, Munich, Germany). The excitation source employed was a Wolfram X-ray tube (continuous spectrum)

at 50 kV and 47 mA. The samples were excited for 100 s and a Si(Li)-detector was used for X-rays acquisition. The samples were prepared by fixating 200 µg of the powders at the center of polished quartz glass reflectors with 5 µL of a 1 % polyvinyl alcohol (PVA) solution and drying on a hot plate for 5 min. Quantification of Mo (in wt.%) was done relative to Ti. The real loadings of $[Mo_3S_{13}]^{2-}$ on TiO_2 were determined considering the stoichiometry of the clusters and the support.

Scanning electron microscopy (SEM) images were acquired using a FEI Quanta 250 FEG scanning electron microscope to obtain visual information on the morphology of the samples. Typically acceleration voltage of 10 kV and secondary electron detection mode were used. High-resolution transmission electron microscopy (HRTEM) images were obtained using FEI TECNAI F20 transmission electron microscope equipped with a field emission gun in bright field mode using 200 kV acceleration voltage. The sample was prepared from powdered samples directly on a copper holey carbon-coated grid (Plano, 200 mesh). Energy-dispersive X-ray spectroscopy (EDS) was performed using scanning TEM (STEM) mode to obtain elemental maps.

High-angle annular dark field (HAADF) STEM imaging and energy dispersive X-ray spectroscopy (EDS) were performed by using Titan Cubed G2 60-300 (TEM/STEM, FEI Co., now Thermo Fisher Scientific) operated at 300 kV. This microscope has an aberration corrector for STEM (DCOR, CEOS), four-quadrant windowless super-X SDD (silicon drift detector) system. The probe current was ~60 pA for STEM observation as well as EDS. The convergence semi-angle of the electron probe was 18 mrad. The typical probe diameter was less than 0.1 nm. Forward scattered electrons of an angular range from 38 to 184 mrad were detected by a HAADF detector for STEM imaging.

Thermogravimetric analysis (TGA) was carried out with a PerkinElmer Thermogravimetric Analyzer TGA 8000. The samples were loaded onto Al_2O_3 crucibles and heated at the rate of 5°C min^{-1} under air and N_2 atmosphere from 25-600°C.

Powder X-ray diffraction (XRD) was performed using an XPERT II: PANalytical XPert Pro MPD (Θ–Θ Diffractometer) for the *ex situ* experiments, and an XPERT III: PANalytical XPert Pro MPD (Θ–Θ Diffractometer) for the *in situ* XRD experiments. The sample was placed on a Si sample holder and irradiated with a Cu X-ray source (8.04 keV, 1.5406 Å). The signals were then acquired with Bragg–Brentano Θ/Θ-diffractometer geometry ranging from 5° to 80° degrees using a semiconductor X'Celerator (2.1°) detector. The *in situ* XRD experiments were performed from 5° to 45° degrees under N_2/air flow through the sample holder, and temperatures ranging from 25 to 800°C.

The physisorption measurements were carried out on a Micromeritics ASAP 2010 by Micromeritics GmbH. The degassing procedure was set to 150-200°C overnight and the isotherms were obtained at 77K using nitrogen as the adsorbate. Surface areas of the supports have been estimated based on the Brunauer–Emmett–Teller (BET) theory.

Steady state photoluminescence (PL) measurements were performed using PicoQuant FluoTime 300 spectrophotometer. Xe arc lamp (300 W power) was the excitation source, coupled with a double-grating monochromator. The detection system comprised of a PMA Hybrid 07 detector along with a high-resolution double monochromator. The excitation wavelength utilized for all the steady state measurements was 315 nm (3.94 eV photon energy). The data was collected and later fitted using EasyTau2 software.

Additional methods

Solubility of the $Na_2[Mo_3S_{13}]$ was checked by preparing 1 mg/mL solutions in water, methanol, ethanol, isopropanol, 1-butanol and phenol. Assisted by light ultrasonication, stable molecular solutions could be obtained in every of these solvent, but 1-butanol and phenol.

$BiVO_4$ was synthesized according to the following procedure. 1 mmol of $Bi(NO_3)3.5H_2O$ were added to 2.5 ml of water, followed by the addition of 100 µl of HNO_3 (67%) – noted as solution A. 1 mmol of NH_4VO_3 were dissolved in 2.5 ml of boiling water– noted as solution B. Solution B was then added dropwise to solution A, forming an orangish-yellow suspension. The pH of the solution was then adjusted to 0.8 using ammonia solution, followed by 15 min of stirring. The suspension was transferred to a 30 ml microwave vial and treated at 150°C for 30 min. After the microwave treatment, the product was washed 3 times with distilled water and ethanol followed by drying at 60°C overnight. XRD confirmed the formation of $BiVO_4$.

Thin-film models systems have been developed on a par with the investigations of powdered Mo_3/TiO_2 samples. For this, original anatase TiO_2 powders were filmed through spin-coating. In a single preparation, 400 mg of TiO_2 was added to a mixture of 2 g of 10 wt.% ethanolic solution of ethyl-cellulose and 1.75 ml of terpineol. The mixture was then sonicated for 30 min followed by stirring for 6 h to obtain a homogenous suspension. 500 µl of this suspension was spin-coated onto a 1 x 3 cm FTO glass at 1500 rpm for 30 sec. The spin-coated samples were then annealed at 550°C for 30 min yielding pure anatase films. The films were then immersed in $Na_2[Mo_3S_{13}]$ solutions overnight following by thorough washing to obtain Mo_3/TiO_2 films. Cluster loading was verified with TXRF (see Characterization techniques in SI) to be 2 wt.%.

Reference Pt/TiO_2 HER experiment was performed by using Pt photodeposition method.[1] While the general HER procedure described in Methods was followed (i.e. 10 mg of TiO_2 was taken as a photocatalyst), the reaction solution was additionally loaded with H_2PtCl_6 aqueous solution to account for 10 wt.% of Pt with regard to the TiO_2 amount to allow for a comparison with the $10Mo_3/TiO_2$ sample.

2. SEM images

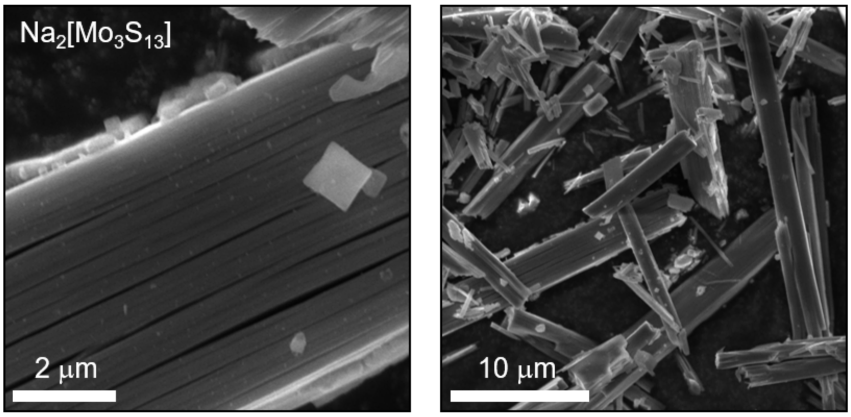

Fig. S1 | Representative SEM images of $Na_2[Mo_3S_{13}]$ powder showing the typical morphology.

3. Additional ATR-FTIR spectra

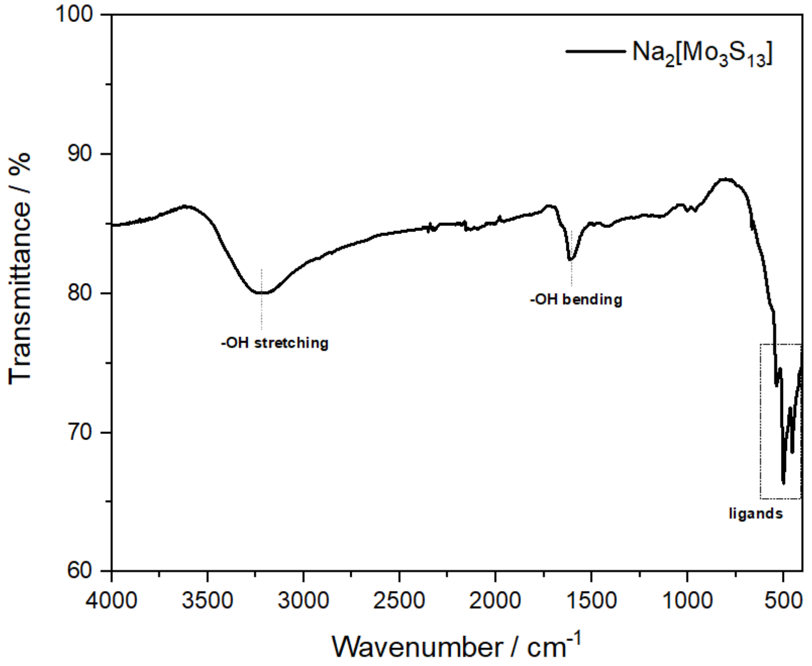

Fig. S2 | ATR-FTIR spectra of the $Na_2[Mo_3S_{13}]\cdot H_2O$ featuring OH bending and stretching vibrations of the water of crystallization. The boxed region containing characteristic S-ligand bands is described in the main text.

4. Additional DRS spectra and analysis

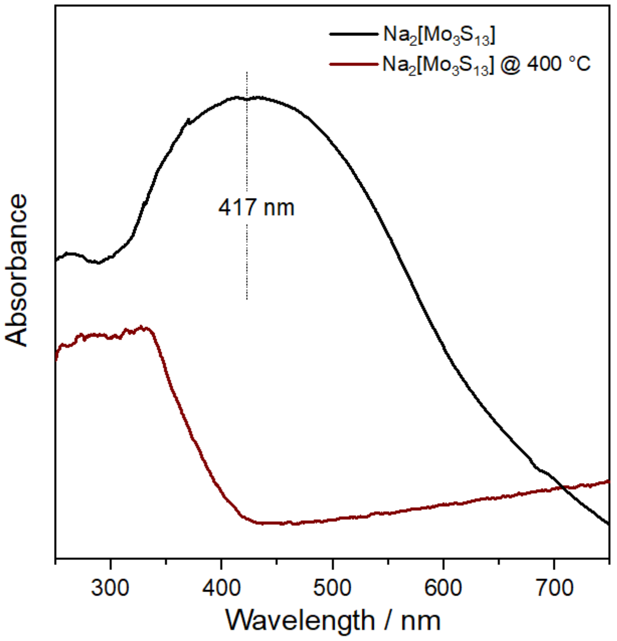

Fig. S3 | DRS spectra of the $Na_2[Mo_3S_{13}]$ and the product of its oxidation in ambient air at 400 °C. The latter product is likely MoO_x featuring an absorption edge corresponding to its band gap. The characteristic $[Mo_3S_{13}]^{2-}$ absorption feature at 417 nm vanishes after oxidation.

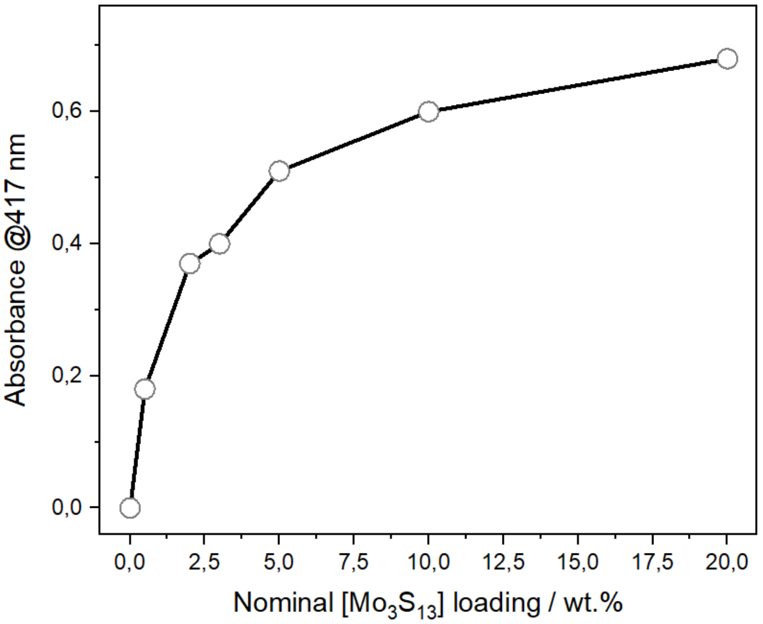

Fig. S4 | Absorption of the Mo_3/TiO_2 composites at 417 nm (after subtracting absorption of TiO_2) plotted against the intended (nominal) cluster loadings. The data correspond to Figure 2b.

5. BET measurements

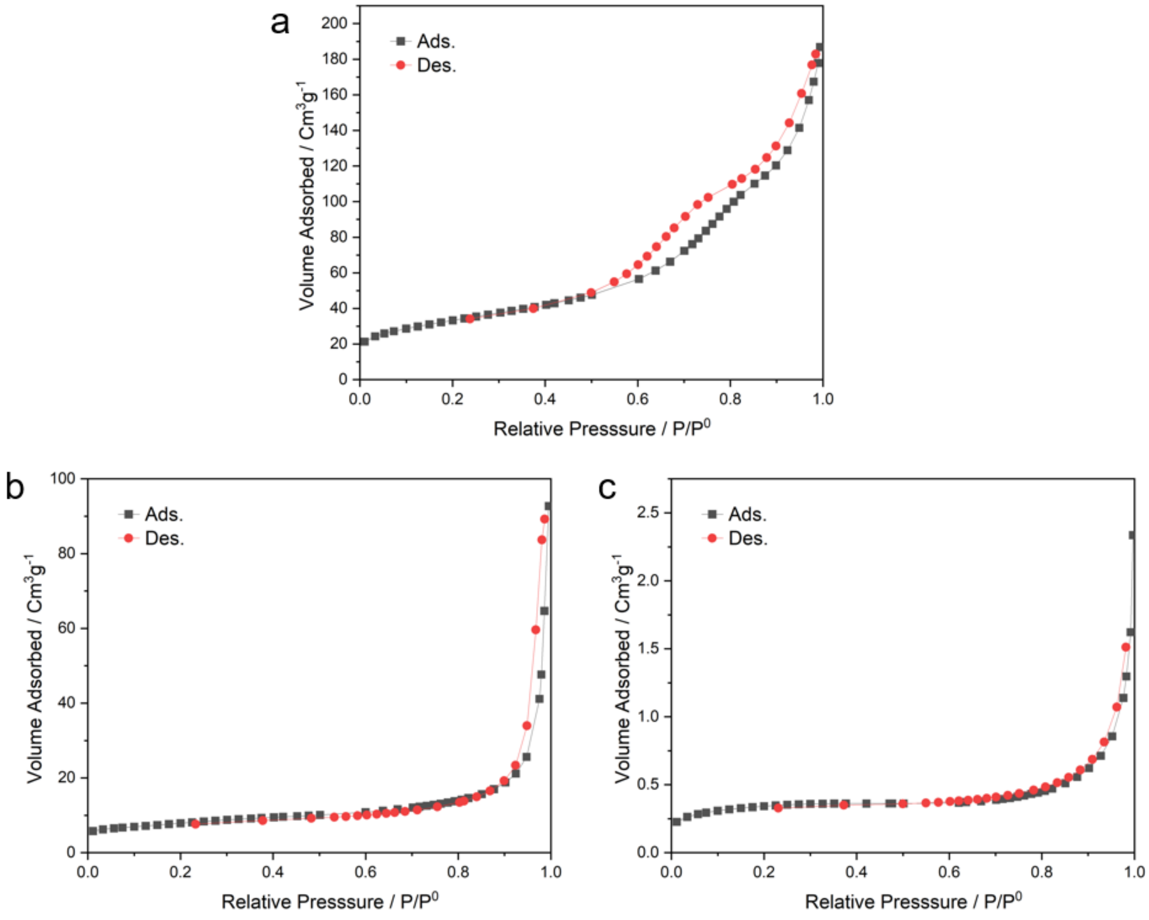

Fig. S5 | N_2 adsorption-desorption isotherms measured for the **(a)** TiO_2 anatase sample originally used as a support, **(b)** commercial rutile TiO_2 and **(c)** home-made $BiVO_4$ powder. Details on the supports are in SI sections of Methods and Chemicals. Values of the BET surface areas for the samples are presented in Table S1.

6. Estimation of the theoretical surface coverage

Using STM analysis, Besenbacher *et al.*[2] found the {Mo_3S_{13}} footprint to be ~ 0.7 nm^2 for a single cluster adsorbed onto the flat surface of highly-oriented pyrolytic graphite. However, this value only corresponds to the geometric area that a free-standing cluster occupies on a flat surface. Based on the solid-state crystal structure of the $(NH_4)[Mo_3S_{13}]$ salt, a single $[Mo_3S_{13}]^{2-}$ cluster in a close-packed lattice takes up around 0.96 nm^2 (see scheme below).

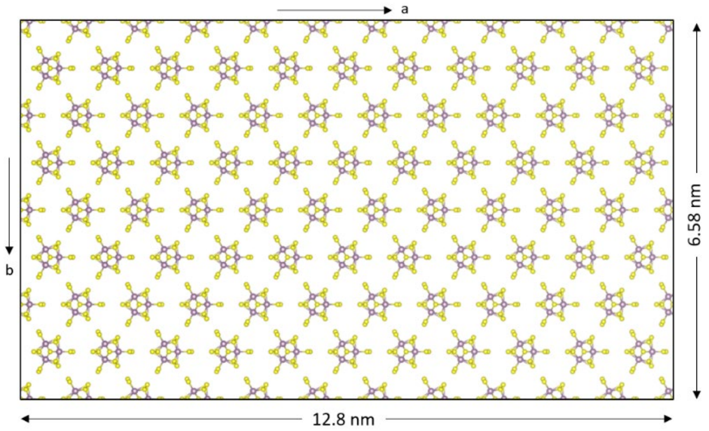

In the case of high coverages of the clusters over the TiO_2 surface in our composites, however, steric and coulombic repulsions of the anionic $[Mo_3S_{13}]^{2-}$ species will contribute to a further spread of the clusters attached to the surface. We estimate the average surface area occupied by a single cluster to increase to around 1.2-1.5 nm^2. From the physisorption data of TiO_2, the BET surface area of the support is around 116 m^2/g. The ratio of the two values gives us the maximum number of clusters that could be loaded onto the TiO_2 surface considering dense monolayer adsorption. Further dividing this value with the Avogadro's number and multiplying by the molecular weight of the cluster (only the anion is considered) gives us the cluster loading (i.e. weight of the cluster on 1 g of TiO_2).

The final calculation yields a loading value range of 8.7 to 10.6 wt.% (average is 9.6 wt.%). This range represents a more realistic scenario when a dense monolayer of $[Mo_3S_{13}]^{2-}$ clusters the entire support surface.

7. Extended XRD patterns

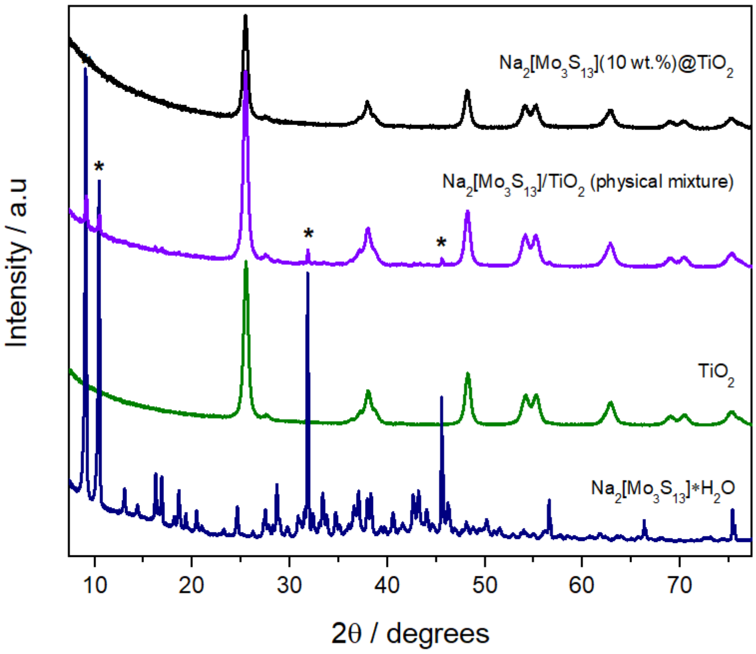

Fig. S6 | Powder XRD patterns of $Na_2[Mo_3S_{13}]$ clusters, anatase-TiO_2, physical mixture of clusters and titania, and Mo_3/TiO_2 composite synthesized by the wet impregnation method.

To elucidate on the state of the $[Mo_3S_{13}]^{2-}$ inside the Mo_3/TiO_2 composites, we performed powder XRD of the $10Mo_3/TiO_2$ sample (real cluster loading of 8.3 wt.%) and compared it to that of a relevant physical mixture of the $Na_2[Mo_3S_{13}]\cdot H_2O$ and TiO_2 (1:9 wt.%).

As Figure S6 shows, XRD spectra of the physical mixture of $Na_2[Mo_3S_{13}]\cdot H_2O$ clusters and TiO_2 exhibits some of the peaks overlapping with the XRD pattern of pure $Na_2[Mo_3S_{13}]$. This corresponds to the presence of thiomolybdate crystals or agglomerates of clusters on the surface of the titania support. Contrary to this, the $10Mo_3/TiO_2$ composite prepared via impregnation shows no peaks corresponding to the XRD pattern of pure $Na_2[Mo_3S_{13}]$. All of the peaks observed are matching well to the XRD pattern of anatase TiO_2 suggesting a good dispersion of the $[Mo_3S_{13}]^{2-}$ clusters over the support surface and the absence of larger cluster aggregates.

8. Additional EDS maps

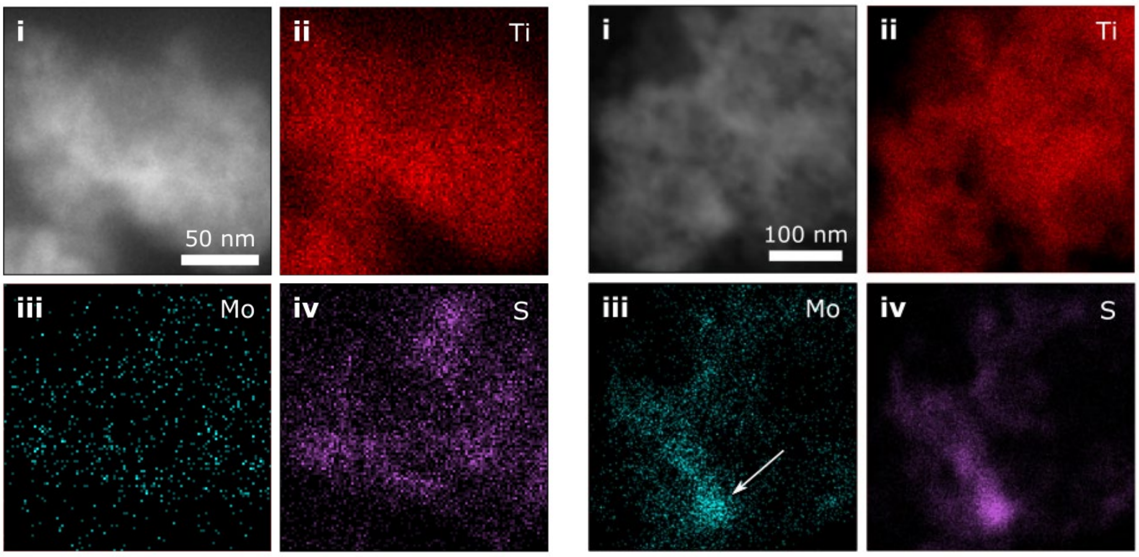

Fig. S7 | Large-area EDS elemental mappings of Ti (ii), Mo (iii) and S (iv) in an exemplary $10Mo_3/TiO_2$ composite. The arrow highlights an area of higher Mo/S concentration. Judging from the lower Ti signal arriving from the area, it can be assumed that the surface of TiO_2 is covered with the MoS_x species.

9. TEM images

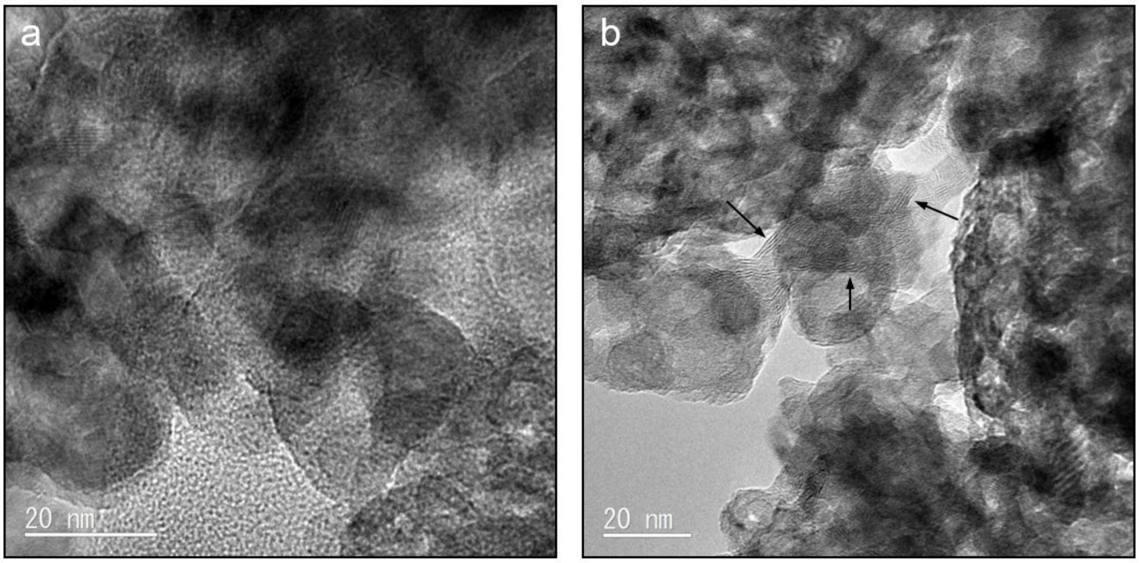

Fig. S8 | **HRTEM images of the exemplary $3Mo_3/TiO_2$ composite. (a)** A collection of TiO_2 nanoparticles corresponding to the original anatase powder (average size between 20 and 30 nm) can be visible, typically no surface aggregates can be observed, **(b)** arrows indicate seldom areas that feature the formation of ordered/layered nanostructures. These areas also correspond to higher elemental contents of S and Mo, suggesting a certain degree of multilayer adsorption and layering. The average interlayer distance is measured to be around 0.7 nm. It is substantially larger of that found in bulk MoS_2 (0.615 nm) and corresponds well to the size of a single $\{Mo_3S_{13}\}$ anion.

10. Deposition of $[Mo_3S_{13}]^{2-}$ on alternative supports

In order to verify the assumption that the cluster deposition follows a monolayer adsorption model (i.e. the maximum loading values are limited by the surface area of the support) and to further elucidate whether this model is only applicable to anatase TiO_2 (i.e. it is specific to its surface chemistry and charge), we performed similar wet-impregnation of three alternative nano- (faceted TiO_2 and rutile TiO_2) and micron-sized ($BiVO_4$) powders using concentrated $Na_2[Mo_3S_{13}]$ solutions that corresponded to 20 wt. % cluster loadings (i.e. mimicking the preparation of $20Mo_3/TiO_2$ described in the main text). In all three cases, after allowing for 24 h adsorption and equilibrium, first washing resulted in strongly red-coloured filtrates, which indicated that a big portion of the original $[Mo_3S_{13}]^{2-}$ clusters has not been anchored strongly to the support surface. TXRF allowed to quantify real $[Mo_3S_{13}]^{2-}$ loadings and revealed support-dependent values in the range between 0.19 and 5.14 wt.% (Table S1). According to BET measurements (Figure S5), all three supports feature low surface areas in the range between 2 and 27 m^2/g (Table S1). When real loadings are plotted against the BET area (Figure S9), a clear trend can be seen. The result is thus in good agreement with the proposed attachment model.

Table S1. Alternative oxide supports used for impregnation, their BET surface areas and the corresponding $[Mo_3S_{13}]^{2-}$ derived from TXRF measurements.

Sample	Maximum $[Mo_3S_{13}]^{2-}$ loading measured by TXRF / wt.%	BET surface area *
$BiVO_4$	0.19	1.11
faceted TiO_2	3.14	13.42 [3]
rutile TiO_2	5.14	27.03

* N_2 physisorption isotherms are presented in Figure S5

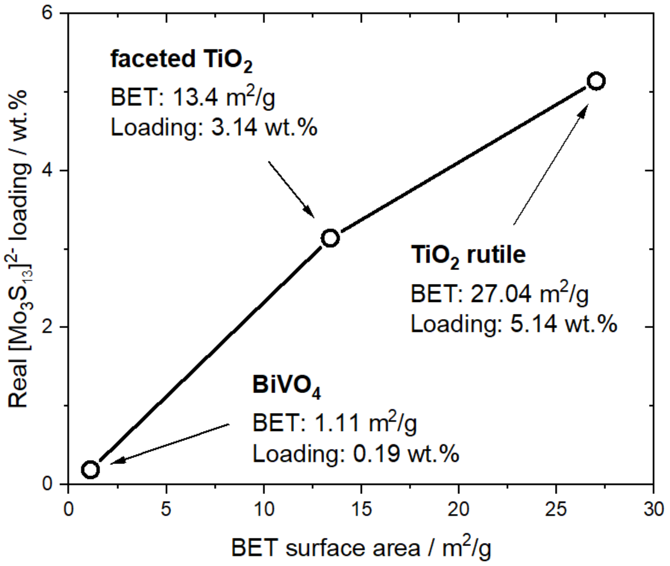

Fig. S9 | Correlation between BET surface areas and maximum cluster loadings achieved for a series of oxide supports.

11. Leaching experiments

The irreversible attachment of $[Mo_3S_{13}]^{2-}$ clusters onto TiO_2 and their stability towards turnover conditions were confirmed by HER leaching experiment. In a single experiment, as-obtained Mo_3/TiO_2 composite was dispersed in methanol/water (1/1) solvent by ultrasonication for 15 seconds. The resulting cloudy solution was then filtered and the filtrate was collected. In order to verify if the filtrate contained any $[Mo_3S_{13}]^{2-}$ detached from the original Mo_3/TiO_2 composite upon ultrasonication, the filtrate solution was charged with 10 mg of anatase TiO_2 nanoparticles. This solution was then stirred for 1 hour to allow for equilibrium (adsorption) and tested for photocatalytic hydrogen evolution reaction using the same procedure followed for normal HER experiments (see Methods section in the main text). The hydrogen evolution performance of so-obtained solution was similar as obtained for bare anatase TiO_2, which suggests that only a negligible amount of $[Mo_3S_{13}]^{2-}$ clusters (< 1 % of the initial amount loaded) leached from Mo_3/TiO_2. This result was further confirmed by analyzing $[Mo_3S_{13}]^{2-}$ loadings of the Mo_3/TiO_2 samples before and after conducting HER tests. Negligible change in Mo contents could be detected.

12. Additional Raman spectra

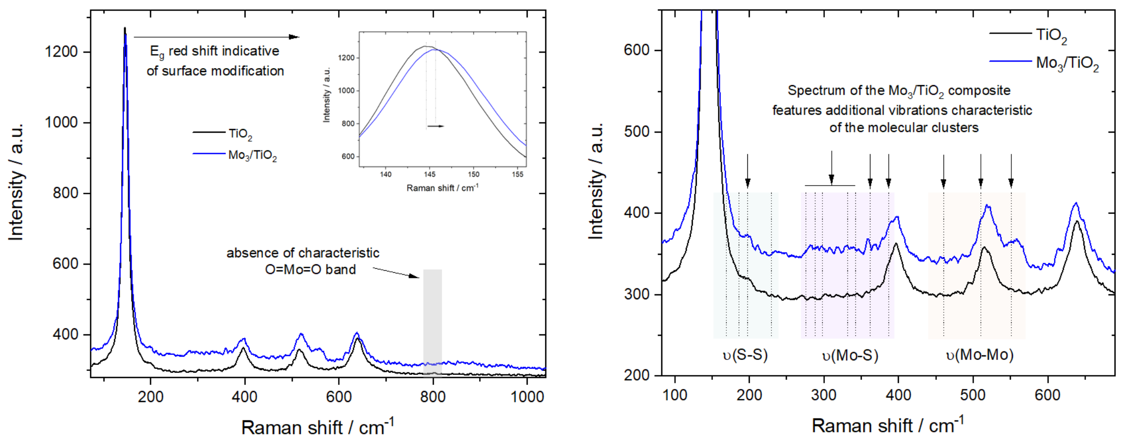

Fig. S10 | **(a)** Raman spectra of $20Mo_3/TiO_2$ composite as well as anatase TiO_2 along with the magnified plot from showing the shift of the TiO_2 E_g band; the absence of Mo=O vibration around 800 cm^{-1} suggests that no oxidation of clusters occurred upon attachment. **(b)** Zoomed-in data with arrows highlighting the features characteristic of the $[Mo_3S_{13}]^{2-}$ cluster.

13. XPS discussion

13.1 XPS quantification

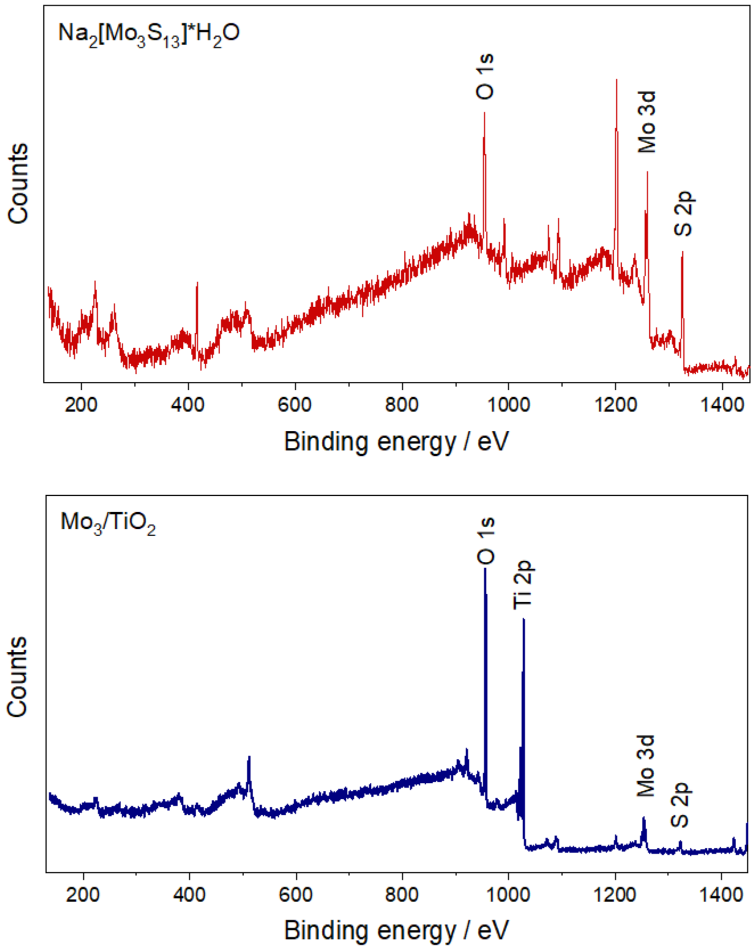

Fig. S11 | XPS survey spectra of **(a)** $Na_2[Mo_3S_{13}]\cdot H_2O$ powder and **(b)** Mo_3/TiO_2 composite (real cluster loading of 2 wt.%) prepared as a thin film, see Methods in SI.

Table S2. Survey quantifications of $Na_2[Mo_3S_{13}]\cdot H_2O$ and Mo_3/TiO_2 samples. Values indicate at.% of the elements derived from the survey spectra quantification.

**Samples**	**Mo 3d**	**S 2p**	**C 1s**	**O 1s**	**Na 1s**	**Ti 2p**
$Na_2[Mo_3S_{13}]\cdot H_2O$	**6.4**	**17.0**	59.6	15.1	2.0	-
Mo_3/TiO_2	**5.0**	**6.5**	53.9	9.9	0.9	23.8

Table S3. Atomic % of S:Mo ratio calculated from XPS data.

**Samples**	**S:Mo**
$Na_2[Mo_3S_{13}]\cdot H_2O$	**2.7**
Mo_3/TiO_2	**1.3**

13.2 XPS discussion

O 1s peak undergoes a considerable shift to higher binding energies upon $[Mo_3S_{13}]^{-2}$ attachment (Figure 3e). This shift is related to the main O component (often called “oxide” component of the “surface bulk” TiO_2, red fitted line) and originally corresponds to Ti-O-Ti binding motif of the titania. Upon the $[Mo_3S_{13}]^{-2}$ attachment, Mo binds to some of the Ti-OH groups forming Ti-O-Mo bonds at the cluster/TiO_2 interface. This results in a new minor “oxide” component (Ti-O-Mo) contributing to the O 1s peak after attachment, which shifts the major Ti-O-Ti peak to higher binding energies due to the higher electronegativity (i.e. lower ability to donate elections) of Mo (2.16) compared to Ti (1.54). Experimentally, we observe a 0.18 eV shift, which aligns well with the surface sensitivity of XPS.

In addition to the Ti-O-M (M stands for Ti or Mo) peak shift, O 1s XPS data also shows a significant drop in the amount of surface-hydroxyls (Figure 3e, blue fitted line) that takes place upon cluster deposition. This observation goes in line with the proposed attachment model, which involves surface Ti-OH groups to covalently bind Mo centers by displacing terminal S_2^{2-} ligands (see discussion in the main text). The decrease of the Ti-O-H signal (ca. 5% based on the relative peak area) corresponds well to their conversion into Ti-O-Mo groups.

Detailed analysis of the XPS data further allows us to clarify the process of charge transfer at the $[Mo_3S_{13}]^{-2}$/TiO_2 interface. The primary evidence for the electron flow is based on Mo 3d XPS data, which suggest that a part of Mo centers get oxidized upon the attachment. We believe that one or two out of three of the Mo atoms in each of the $[Mo_3S_{13}]^{-2}$ clusters – primarily those that undergo covalent binding with O atoms of the surface – change their oxidation state from +4 to +5/+6 depending on the binding scenario. This oxidation implies electron flow across the interface i.e. charge transfer. Besides this, a consistent strong (ca. 1 eV) shift of Mo XPS peaks to higher binding energy (Figure 3c) also implies a general displacement of electron density from the cluster. This may be related to the overall negative charge of the $[Mo_3S_{13}]^{-2}$ anion, which in this case acts as a strong electron donor. What is interesting, however, we do not observe any simultaneous XPS shift of Ti 2p or O 1s to lower binding energy. In contrary, as discussed before, O 1s shifts to higher binding energy (although, to a far lower extent, 0.18 eV) the reason for which was discussed above. Overall, this situation allows us to conclude that the charge transfer from the cluster to the support is in place, however, the transferred electrons do not localize at the (sub-)surface of TiO_2, but rather tend to dissipate into the bulk.

14. Supplementary photocatalytic performance data

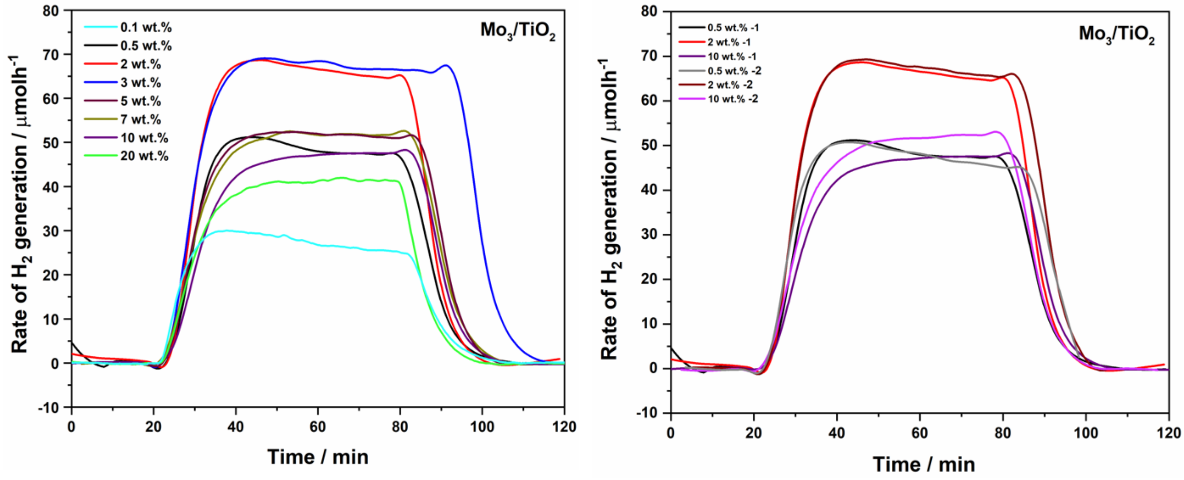

Fig. S12 | **(a)** The rate of hydrogen generation in μmol/h displayed by Mo_3/TiO_2 composites with 0.1-20 wt.% of cluster loading, **(b)** Reproducibility test for photocatalytic HER performance of Mo_3/TiO_2 composites with 0.5, 2, and 10 wt.% of cluster loading. Conditions: 0.25 mg/mL photocatalyst in MeOH:H_2O (1:1) illuminated by 365 nm LED source for 1 hour; see Methods for details. The photocatalytic performance plot in the main manuscript was constructed using the activity value reached after 60 minutes of light illumination.

In order to validate our photocatalytic setup, we performed blank and reference experiments, including those without light, without Mo_3/TiO_2 catalyst, using neat TiO_2 and neat $[Mo_3S_{13}]^{-2}$ cluster solution. In all the cases, we observed no H_2 evolution, which demonstrates that Mo_3/TiO_2 serves as an active HER photocatalyst.

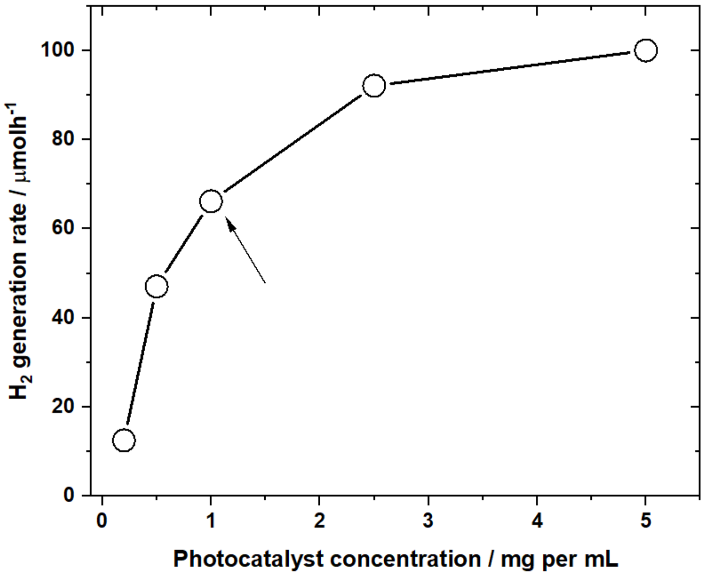

Fig. S13 | The rate of H_2 evolution plotted as a function of photocatalyst amount used.[4] Standard conditions used in this work correspond to 1 mg/mL suspensions of the $2Mo_3/TiO_2$ powders (details in Methods section of the main text). At this concentration, we ensure that sufficient photon flux is available for the absorption by the TiO_2, which allows minimizing the impact of $[Mo_3S_{13}]^{2-}$ parasitic absorption on the obtained H_2 generation rates.

15. Post-catalytic characterization

15.1 TXRF and EDS data

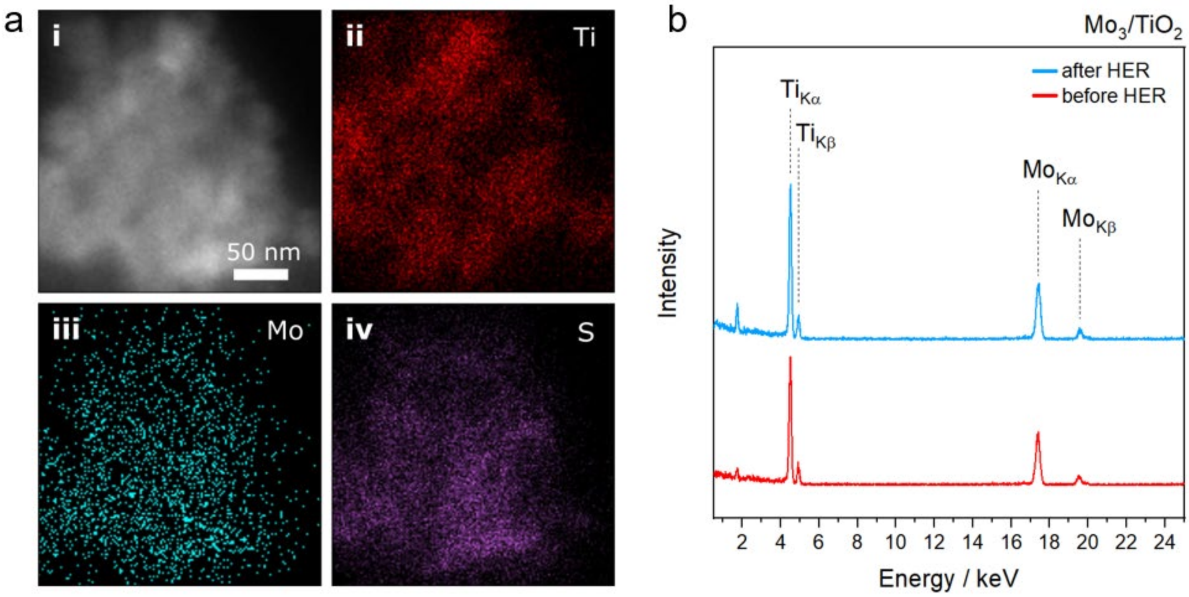

Fig. S14 | **(a)** EDS elemental mappings of Ti (ii), Mo (iii) and S (iv) of an exemplary $3Mo_3/TiO_2$ composite after the HER run showing the presence and homogeneous distribution of the relevant elements. **(b)** TXRF spectra of the Mo_3/TiO_2 before and after HER run showing the unchanged content of Mo.

Quantification of the TXRF data (see SI section Characterization techniques) and relative Ti:Mo ratios reveals cluster loading values of 5.06 wt.% (before reaction) and 4.90 wt.% (after reaction), which suggest that only a minor amount of leaching took place over the course of the photocatalytic reaction.

15.2 XPS data

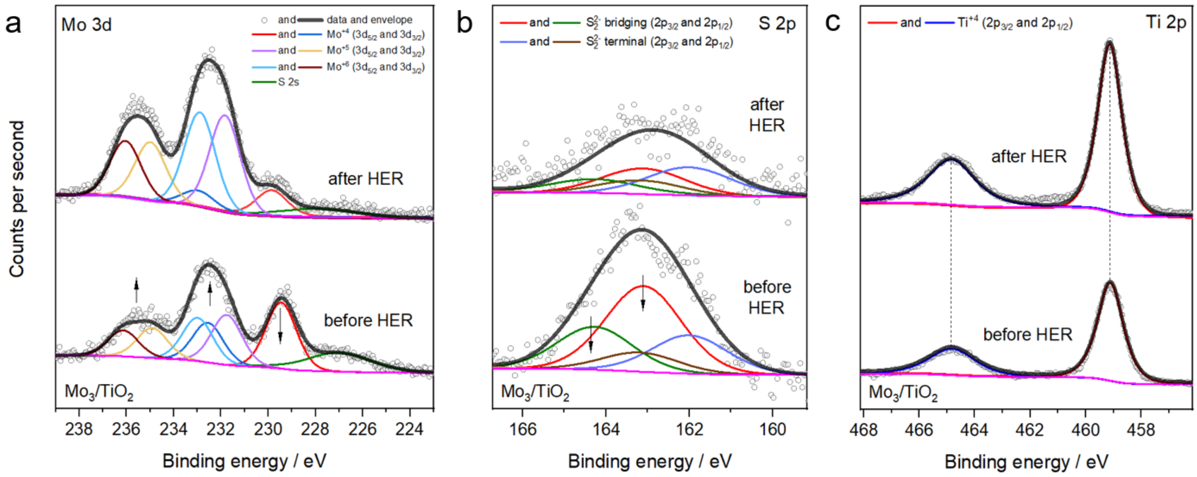

Fig. S15 | XPS spectra of the Mo_3/TiO_2 composites before and after HER run showing **(a)** Mo 3d, **(b)** S 2p and **(c)** Ti 2p regions with relevant fits.

16. Thermal treatments (TGA and *in situ* XRD) and HER performance

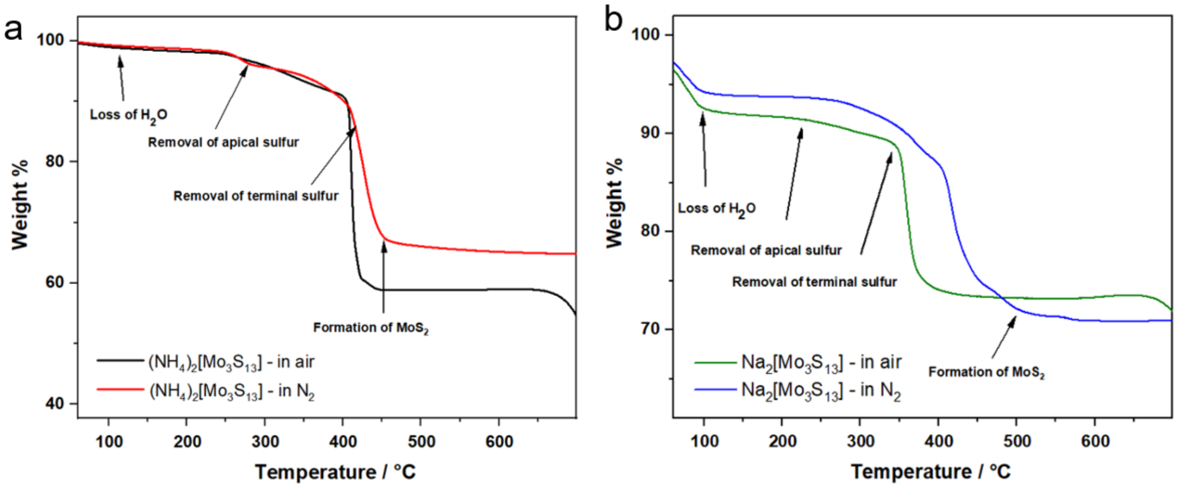

Fig. S16 | TGA profiles of **(a)** $(NH_4)_2[Mo_3S_{13}]$ and **(b)** $Na_2[Mo_3S_{13}]$ powders – used here to represent the behavior of $[Mo_3S_{13}]^{2-}$ anion – obtained under air (black and green) and N_2 (red and blue). Oxidation (in air) or transformation (in N_2) of the $[Mo_3S_{13}]^{2-}$ clusters take place in the temperature window of 250-450 °C. Decomposition of the cluster is facilitated in air compared to N_2.

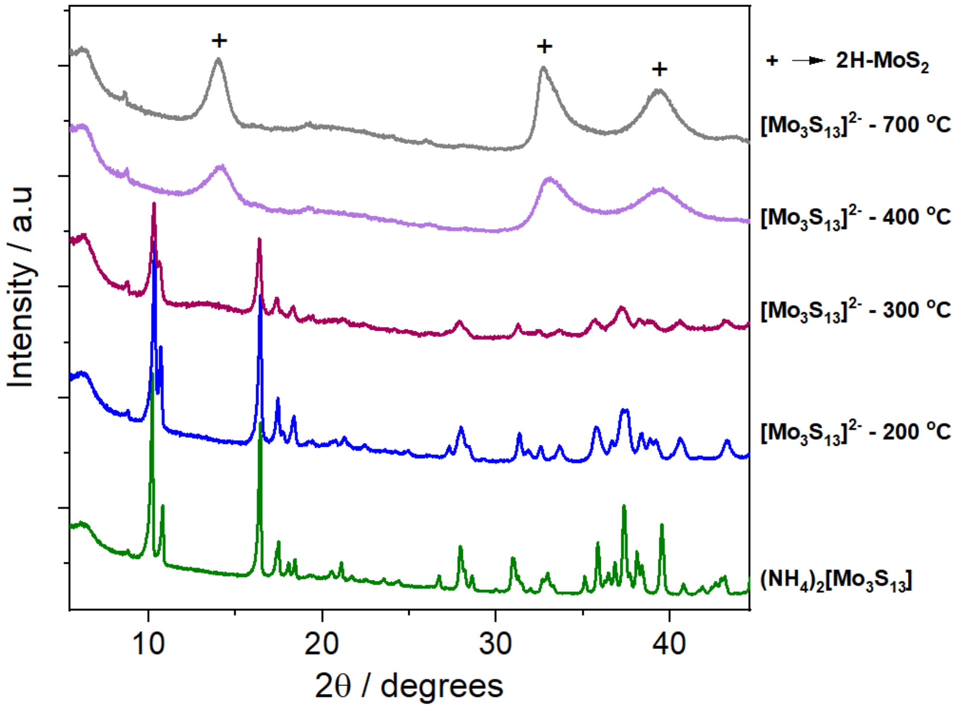

Fig. S17 | *in situ* XRD patterns obtained upon $(NH_4)_2[Mo_3S_{13}]$ – used here to represent the behavior of $[Mo_3S_{13}]^{2-}$ anion – heating under inert N_2 atmosphere (see Characterization techniques). The intact structure of the $[Mo_3S_{13}]^{2-}$ can be observed till at least 300 °C. Transformation to MoS_2-like pattern (broad peaks indicate small areas of structural order) takes place within the temperature window of 300-400 °C. Note: in situ XRD datasets of the $Na_2[Mo_3S_{13}]$ are more convoluted and were thus omitted for clarity.

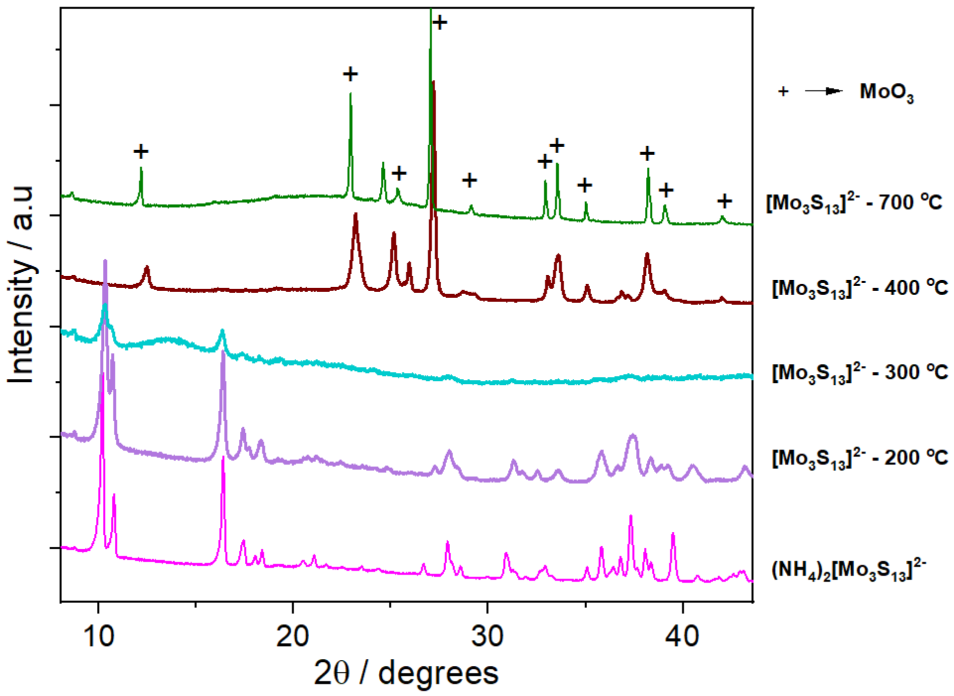

Fig. S18 | *in situ* XRD patterns obtained upon $(NH_4)_2[Mo_3S_{13}]$ heating under ambient air. The intact structure of the $[Mo_3S_{13}]^{2-}$ can be detected till at least 200 °C. In contrast to the treatment in N_2 (see above), XRD pattern at 300 °C manifests a strong loss of crystallinity, likely related to the facilitated oxidation and transformation of the original structure. The appearance of MoO_3 pattern takes place within the temperature window of 300-400 °C.

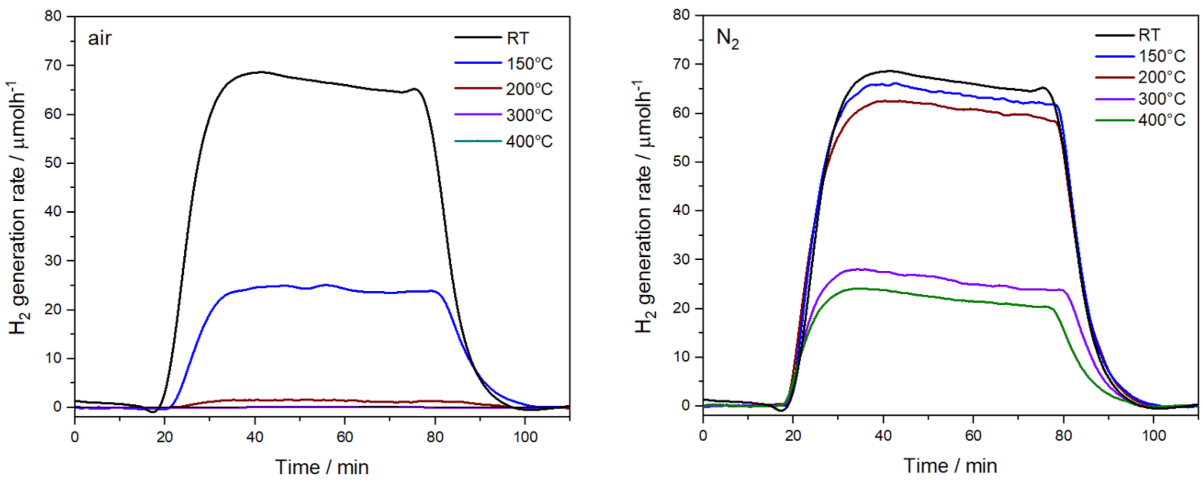

Fig. S19 | The hydrogen evolution rate in μmol/h displayed by $2Mo_3/TiO_2$ photocatalyst heat-treated at different temperatures (150-400 °C) in **(a)** air and **(b)** N_2.

17. List of references

(1) Wenderich, K.; Mul, G. Methods, Mechanism, and Applications of Photodeposition in Photocatalysis: A Review. *Chem. Rev.* **2016**, *116* (23), 14587–14619. https://doi.org/10.1021/acs.chemrev.6b00327.

(2) Kibsgaard, J.; Jaramillo, T. F.; Besenbacher, F. Building an Appropriate Active-Site Motif into a Hydrogen-Evolution Catalyst with Thiomolybdate $[Mo_3S_{13}]^{2-}$ Clusters. *Nat. Chem.* **2014**, *6* (3), 248–253. https://doi.org/10.1038/nchem.1853.

(3) Kashiwaya, S.; Olivier, C.; Majimel, J.; Klein, A.; Jaegermann, W.; Toupance, T. Nickel Oxide Selectively Deposited on the {101} Facet of Anatase TiO2 Nanocrystal Bipyramids for Enhanced Photocatalysis. *ACS Appl. Nano Mater.* **2019**, *2* (8), 4793–4803. https://doi.org/10.1021/acsanm.9b00729.

(4) Qureshi, M.; Takanabe, K. Insights on Measuring and Reporting Heterogeneous Photocatalysis: Efficiency Definitions and Setup Examples. *Chem. Mater.* **2017**, *29* (1), 158–167. https://doi.org/10.1021/acs.chemmater.6b02907.
